# Cost-effectiveness of the sFlt-1/PlGF ratio and telemonitoring in managing suspected pre-eclampsia: protocol for the PREPARE II randomised controlled trial

**DOI:** 10.1136/bmjopen-2025-113516

**Published:** 2026-07-02

**Authors:** M Wind, A Peters, M E van den Akker-van Marle, K Vanden Auweele, L van Bodegom-Vos, F Boekhorst, C M Cobbaert, W Hermes, M E Van Hoorn, L Van Den Haak, Margo Lutke Holzik, R Tsonaka, L Van Wyk, Y K O Teng, M Sueters

**Affiliations:** 1 Department of Obstetrics, Leiden University Medical Center, Leiden, Zuid-Holland, the Netherlands; 2 Department of Biomedical Data Sciences, Leiden University Medical Center, Leiden, Zuid-Holland, the Netherlands; 3 Patient’ Association, The Hellp Foundation, Zwolle, Overijssel, the Netherlands; 4 Department of Obstetrics, Groene Hart Hospital, Gouda, Zuid-Holland, the Netherlands; 5 Department of Clinical Chemistry, Leiden University Medical Center, Leiden, Zuid-Holland, the Netherlands; 6 Department of Obstetrics, Haaglanden Medical Center, The Hague, Zuid-Holland, the Netherlands; 7 Department of Obstetrics, Haga Hospital, The Hague, Zuid-Holland, the Netherlands; 8 Department of Obstetrics, Reinier de Graaf Hospital, Delft, Zuid-Holland, the Netherlands; 9 Department of Obstetrics, Alrijne Hospital, Leiden, Zuid-Holland, the Netherlands; 10 Department of Nephrology, Leiden University Medical Center, Leiden, ZH, the Netherlands

**Keywords:** Maternal medicine, Hospitalization, Hypertension, Pregnant Women, Health Care Costs

## Abstract

**Introduction:**

The soluble FMS-like tyrosine kinase-1 and placental growth factor (sFlt-1/PlGF) ratio has demonstrated impressive predictive test characteristics in women with suspected pre-eclampsia. However, it remains a matter of debate whether the introduction of this novel test can indeed translate to a reduction in pre-eclampsia-related hospital admissions, outpatient visits and can consequently lower overall healthcare costs. The PREPARE II study aims to investigate whether the sFlt-1/PlGF ratio, along with digital self-monitoring, can reduce pre-eclampsia-related healthcare utilisation in the first week following the test for women with suspected pre-eclampsia.

**Methods and analysis:**

This is a randomised controlled trial across six centres which includes women (≥16 years old) between 20 and 37 weeks of gestation with suspected pre-eclampsia due to one or more identified symptoms. For power calculation, we assumed that the sFlt-1/PlGF ratio including a telemonitoring strategy leads to a de-escalation of care by cumulatively reducing the frequency of pre-eclampsia-related hospital admissions and/or outpatient visits in the first week from 50% in the control group to 35% in the intervention group. Considering a loss to follow-up of 5%, a sample size of approximately 470 women is required with 235 women per arm (α=5%; power=90%). The intervention is an algorithm based on the urine protein/creatinine ratio (PCr)+sFlt-1/PlGF ratio, along with a telemonitoring strategy. The algorithm incorporates a PCr cut-off of 30 (mg/mmol) and a sFlt-1/PlGF ratio cut-off of 38 for risk classification. Subsequent clinical follow-up recommendations are stratified based on this classification: low risk entails no additional follow-up, returning to routine antenatal care; intermediate risk includes telemonitoring; high risk necessitates immediate admission. The primary outcome is the occurrence of pre-eclampsia-related healthcare utilisation in the first week after testing. Secondary outcomes are maternal/perinatal adverse events, total healthcare usage, pre-eclampsia diagnosis, quality of life and productivity losses. A cost-effectiveness analysis from a societal perspective will be performed.

**Ethics and dissemination:**

Ethical approval was obtained from the Medical Ethics Committee of Leiden University Medical Centre (METC LDD) on 21 July 2025 (reference NL-009295). The results will be disseminated through peer-reviewed publications and presentations at international conferences.

**Trial registration:**

NL88527.058.24.

**STRENGTHS AND LIMITATIONS OF THIS STUDY**

- A predefined risk stratification algorithm based on the protein-to-creatinine ratio and soluble FMS-like tyrosine kinase-1 and placental growth factor ratio is used to standardise clinical decision-making across study sites.
- Telemonitoring is incorporated into follow-up using predefined thresholds for clinical review.
- A comprehensive economic evaluation from a societal perspective is included, covering healthcare costs, quality of life and productivity losses.
- The study is powered for healthcare utilisation rather than rare maternal or perinatal outcomes.
- Treating clinicians may deviate from the protocol based on clinical judgement, potentially introducing variability in management.

## Introduction

 Pre-eclampsia remains a significant global health concern, affecting 3%–5% of pregnancies and posing both immediate and long-term cardiovascular risks to mothers, as well as significant risks to the unborn child.^1^,^5^ Pre-eclampsia is a syndrome or collection of symptoms to identify pregnant women at risk of developing serious and even life-threatening complications such as seizures, intracranial haemorrhage, pulmonary oedema, hepatic haematoma/rupture, acute kidney injury, coagulopathy, placental abruption, fetal growth restriction and fetal death. Therefore, women presenting with symptoms leading to suspicion of pre-eclampsia often require hospitalisation and/or intensive monitoring.^2 6^

However, the current diagnostic procedures in standard care which involve blood pressure measurements and urine protein-to-creatinine ratio (PCr), inadequately identify pregnant women at risk of developing pre-eclampsia and associated complications.^7 8^ As a result, women with symptoms linked to pre-eclampsia may undergo unnecessary hospitalisation and intensive monitoring until pre-eclampsia is ruled out. These admissions, along with unforeseen additional visits to the outpatient clinic in addition to routine antenatal visits, impose a substantial burden on pregnant women and contribute to potentially avoidable high healthcare costs.^9 10^

Recent studies exploring soluble FMS-like tyrosine kinase-1 (sFlt-1) and placental growth factor (PlGF) levels in pregnant women suggest potential value in predicting the absence of pre-eclampsia.^11 12^ The PROGNOSIS study demonstrated that a sFlt-1/PlGF ratio of <38 had a negative predictive value of 99.3% for ruling out development of pre-eclampsia within the next week.^11^ Ruling out the development of pre-eclampsia for a certain time period in women suspected of the disease may lead to a reduction in over-diagnosis, redundant admission, over-treatment and will consequently lower the costs.^12^ Notwithstanding the impressive test characteristics, studies like INSPIRE, PRERISK, PARROT-UK/Ireland have shown minimal changes in hospital admissions and complication rates, emphasising that the efficacy of this novel test in a real-world setting remains debatable.^13^,^16^

In the PREPARE I study, the sFlt-1/PlGF ratio was investigated in the setting of daily clinical care in the Netherlands which includes routine testing for urine PCr in women with suspected pre-eclampsia.^17^ This study demonstrated the sFlt-1/PlGF ratio in addition to PCr may lead to improved selection of women at risk and a reduction of hospital care without compromising the safety of mothers and neonates. This raises the hypothesis that a more refined selection of women at risk for complications should be accompanied by a corresponding de-escalation of care. Therefore, we have designed the PREPARE II multicentre randomised controlled trial investigating the PCr+sFlt-1/PlGF ratio including a telemonitoring strategy for women with suspected pre-eclampsia.

### Study objectives

Primary objective: to assess whether risk-stratification using the PCr + sFlt-1/PlGF ratio including a telemonitoring strategy reduces pre-eclampsia-related healthcare utilisation (defined as pre-eclampsia-driven admissions and outpatient visits) during the first week after testing in women with suspected pre-eclampsia.

Secondary objective: to investigate if the introduction of the PCr + sFlt-1/PlGF ratio including a telemonitoring strategy for women with suspected pre-eclampsia leads to a favourable cost-effectiveness ratio. Secondary outcomes will be assessed by the investigator up to 6 weeks postpartum.

## Methods and analysis

### Study design

**Figure 1:**
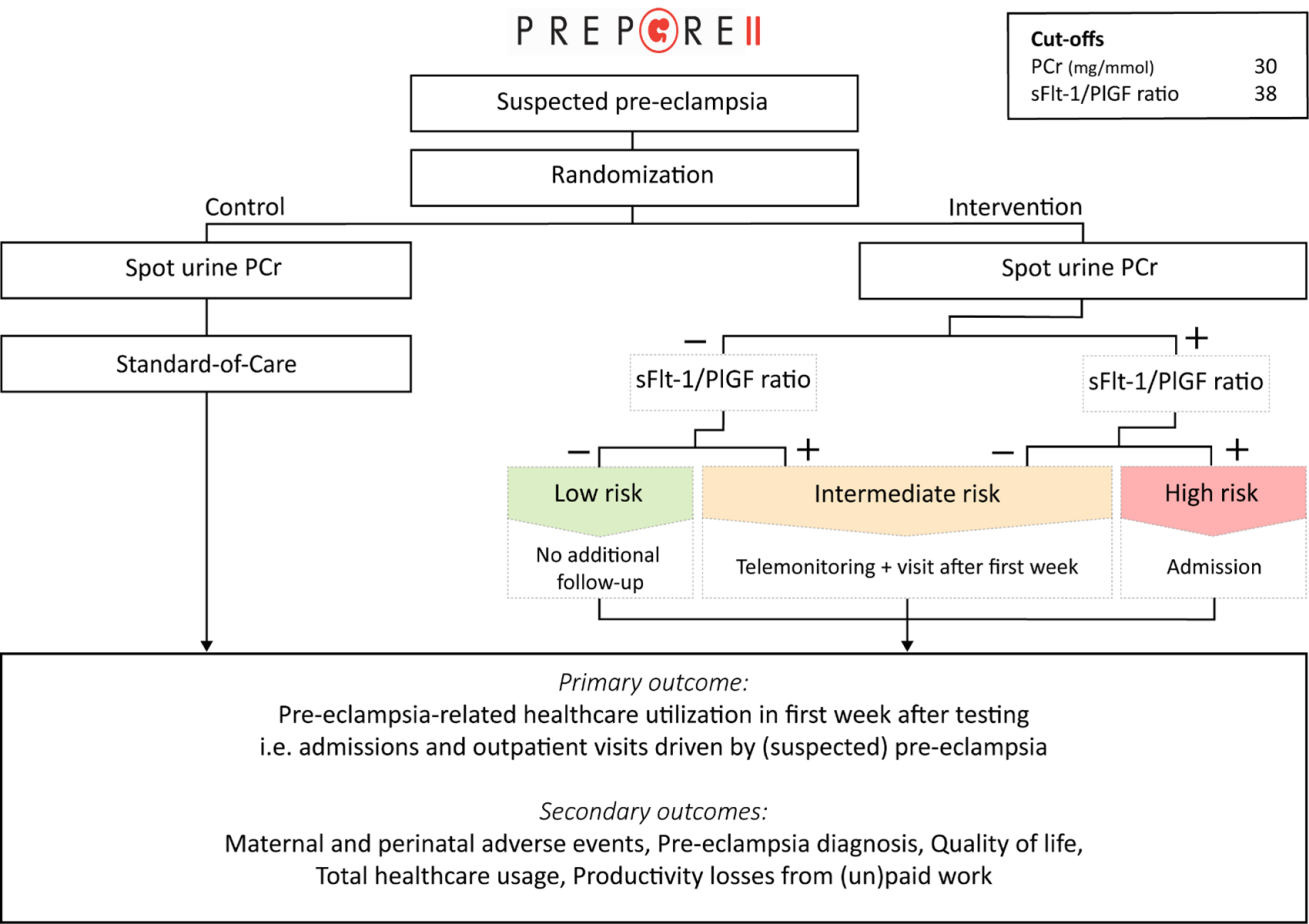
Study design PREPARE II. Women with suspected pre-eclampsia are randomised at first presentation in the control group following standard-of-care or the intervention arm. Follow-up in the intervention arm is determined for each group based on this flow chart: low risk: no additional follow-up; intermediate risk: telemonitoring + weekly visit; high risk: direct admission. PCr, protein-to-creatinine ratio; sFlt-1/PlGF, soluble FMS like tyrosine kinase 1/placenta growth factor.

The PREPARE II study is a randomised controlled trial including a cost-effectiveness analysis across six centres: Leiden University Medical Centre (LUMC), Groene Hart Hospital, Haaglanden Medical Centre, Haga Hospital and Alrijne Hospital and Reinier de Graaf Hospital. Randomisation will be performed on patient level. We expect to reach the target sample size of 470 participants within 4 years. A schematic overview of the study design is shown in figure 1.

### Eligibility criteria

The study population consists of pregnant women with suspected pre-eclampsia. Inclusion and exclusion criteria are shown in box 1.

Box 1 **. Inclusion and exclusion criteria**

Subjects enrolled in the study must meet the following inclusion criteria:

1. Age ≥ 16 years old
2. Singleton pregnancy
3. Gestational age ≥ 20 weeks + 0 days and < 37 weeks + 0 days
4. Suspected pre-eclampsia as defined per protocol, one or more of following:

a. New onset of elevated blood pressure

- Systolic blood pressure ≥140 mmHg and/or
- Diastolic blood pressure ≥90 mmHg

b. Aggravation of pre-existing hypertension

c. New onset of protein in urine

- Proteinuria detected by dipstick >2+

d. Aggravation of pre-existing proteinuria

- Increase of >50% in protein-to-creatinine ratio compared to previous measurement

e. One or more other reason(s) for clinical suspicion of pre-eclampsia:

- Epigastric pain
- Severe oedema or sudden swelling of face, hands, or feet
- Headache or visual disturbances (e.g., blurred vision, scotomas)
- Sudden weight gain >1 kg/week (in third trimester)
- Thrombocytopenia (platelet count <150 × 10⁹/L)
- Elevated liver enzymes (ALT >41 U/L or AST >40 U/L)
- (Suspected) fetal growth restriction (FGR) based on clinical or ultrasound findings

Subjects are excluded from participation if they meet any of the following exclusion criteria:

1. Confirmed pre-eclampsia^2^ at the time of enrolment.
2. Any sFlt-1/PlGF ratio measured this pregnancy.
3. Previous participation in the PREPARE II study in current or prior pregnancy.
4. Gestational age ≥37 weeks.
5. Multiple pregnancy.
6. Insufficient understanding of Dutch or English language.
7. Inability to give informed consent.

### Procedures and randomisation

Women who present with clinical signs consistent with pre-eclampsia between 20 and 37 weeks of gestation at the obstetric ward or outpatient clinic will be screened for eligibility and invited to participate. Eligible individuals will receive standardised verbal and written study information. The participant informed consent form is provided as online supplemental material. If informed consent is provided, it will be obtained within 1 hour of presentation to enable timely clinical decision-making, particularly in the intervention arm. After informed consent is obtained, participants will be randomised through Castor EDC (CDMS 2025 2.3.0), a secure web-based system, in a 1:1 ratio to either the intervention or control arm. Randomisation will be performed using variable block sizes (4, 6 and 8) to ensure allocation concealment, with stratification by centre of inclusion and gestational age (<34 weeks or ≥34 weeks). Due to the nature of the intervention, neither treating clinicians nor participants can be blinded. Trial allocation and the rationale for inclusion will be documented in the participant’s electronic health record. A venous blood sample (20 mL) will be collected from both control and intervention groups, and sent to the clinical chemistry laboratory in Leiden via lab transport. Samples from the intervention group will be transported via emergency/private transport, while samples from the control group will be sent via routine laboratory transport. On availability of the test result, the treating clinician will be notified by the clinical chemistry department only if the participant is allocated to the intervention arm. Throughout the period until the test result is obtained (with a maximum duration until next morning), participants will adhere to standard-of-care (SoC) regardless of their study arm allocation.

### Interventions

#### Intervention arm

In the intervention arm, risk classification is based on a protocolised algorithm involving PCr and sFlt-1/PlGF ratio, along with a telemonitoring strategy (box 2). The algorithm incorporates a PCr cut-off of 30 (mg/mmol) and a sFlt-1/PlGF ratio cut-off of 38 with subsequent clinical follow-up recommendations as shown in box 2: low risk entails no additional follow-up, returning to routine antenatal care; intermediate risk includes telemonitoring; and high risk necessitates immediate admission.

Box 2 **Risk classification and follow-up in the intervention arm**

**Risk classification**

- Low = PCr <30 and sFlt-1/PlGF ratio ≤38.
- Intermediate = PCr <30 and sFlt-1/PlGF ratio >38 or PCr ≥30 and sFlt-1/PlGF ratio ≤38.
- High = PCr ≥30 and sFlt-1/PlGF ratio >3.

Clinical follow-up recommendations are based on risk classification:*

- Low = no additional follow-up/return to routine antenatal care.
- Intermediate = telemonitoring + visit to outpatient clinic after first week.
- High = direct admission.

*If necessary, the treating clinicians can always deviate from study protocol based on their own expert opinion or patient’s presentation.

PCr, protein/creatinine ratio; PlGF, placental growth factor; sFlt-1, soluble FMS-like tyrosine kinase-1.

The most important co-intervention of this study is telemonitoring, which is a digital platform enabling home blood pressure measurements and pre-eclampsia symptoms reporting, in line with the SAFE@HOME study.^18^ For telemonitoring, pregnant women are instructed to perform blood pressure measurements and report symptoms (headache, visual symptoms, epigastric pain, swelling, etc). The digital platform generates automated alerts when blood pressure readings exceed 140/90 mm Hg or when reported symptoms suggest possible pre-eclampsia. Investigators review participant data on a daily basis and communicate findings to the responsible obstetrician. In the event of abnormal or concerning signals, the obstetrician provides clinical management advice. If disease deterioration is suspected, the participant is referred without delay to the outpatient clinic for further evaluation. Following the initial week of telemonitoring, the decision to continue this form of monitoring will be at the discretion of the treating physician. If deemed necessary, treating physicians are permitted to deviate from the study protocol, based on their professional judgement. Such deviations from the protocol are not considered part of the primary or secondary endpoints of the study. However, they will be documented and reported to evaluate the percentage of protocol violation.

#### Control arm

Women in the control arm will receive SoC follow-up according to physician’s discretion, including outpatient clinic visits and admission if needed. Clinical evaluation will include blood pressure measurements, urine PCr, and laboratory testing, with management guided by the ISSHP 2018 criteria.^1^ The sFlt-1/PlGF ratio is not revealed to the clinician in the control arm.

### Study outcomes

#### Main study parameter/endpoint

- Pre-eclampsia-related healthcare utilisation in first week after testing, defined as:
  - Pre-eclampsia-related admissions defined as admissions driven by suspected pre-eclampsia or pre-eclampsia in differential diagnosis as documented by the physician in a structural questionnaire when a patient is admitted.
  - Pre-eclampsia-related outpatient visits defined as a visit to the outpatient clinic driven by suspected pre-eclampsia or pre-eclampsia in differential diagnosis in addition to the routine antenatal visits as documented by the physician.

#### Secondary study parameters/endpoints

- Actual development of pre-eclampsia according to the classification criteria of the ISSHP 2018 definition.^2^
- Composite of maternal adverse outcomes.
  - The occurrence of death, stroke, eclampsia, blindness, hypertension requiring administration of intravenous antihypertensives, the use of inotropic agents, thromboembolic events (arterial, venous or small vessel thrombosis, other than superficial venous thrombosis, in any tissue or organ), pulmonary oedema (diagnosed clinically with one or more of oxygen saturation <95%, diuretic treatment or X‐ray confirmation), respiratory failure (needing intubation), myocardial ischaemia or infarction, hepatic dysfunction (leading to disseminated intravascular coagulation), hepatic haematoma or rupture (confirmed by imaging or at laparotomy), renal failure (serum creatinine >200 µmol/L) and transfusion of any blood products. One patient can have more than one adverse outcome. We will report on the frequency of pregnant women encountering at least one adverse outcome.
- Composite of perinatal adverse outcomes.
  - Preterm delivery (spontaneous and iatrogenic before 37 and 32 weeks), fetal growth restriction (birthweight <10th percentile), admission to the neonatal intensive care unit and perinatal death. One newborn can have more than one adverse outcome. We will report on the frequency of newborns encountering at least one adverse outcome.
- Change in quality of life (EuroQol 5-Dimension 5-Level (EQ-5D-5L))
- Total healthcare usage (ie, admissions, home-monitoring, telemonitoring and outpatient visits beyond first week after baseline).
- Productivity losses from paid and unpaid work (iMTA Productivity Cost Questionnaire (iPCQ)*)*.

A schematic overview of the time of enrolment, interventions and assessments is shown in table 1.

**Table I:** Schedule of enrolment, interventions and assessments in PREPARE II study

		Study period				
**Timepoints**	Presentation at outpatient clinic with suspected PE	Baseline and randomisation	First week after baseline	Every 2 weeks till delivery	Delivery (in hospital or at home)	Close out visit 6 weeks postpartum
Enrolment						
Eligibility screen	X					
Informed consent	X					
Allocation		X				
Interventions (in-person)						
sFlt-1/PlGF measurement		X				
Follow-up decision after 24 hours		X				
No follow-up/telemonitoring/admission			X			
Questionnaire (EQ5D-5L-, iPCQ)		X		X		X
Assessments (charts)						
Demographics		X				
History, comorbidities		X				
Physical measurements		X				
Clinical readings		X				
Maternal outcomes					X	X
Perinatal outcomes					X	X
Prenatal hospital admissions						X
Outpatient visits						X

### Sample size calculation

Using the primary outcome of pre-eclampsia-related healthcare utilisation in the first week after testing, we based our assumptions on the previously conducted PREPARE I study.^17^ In this study in SoC, 50% of the women with suspected pre-eclampsia were either admitted, seen at the outpatient clinic or assigned to home monitoring in the first week after baseline. In test scenario 2, consistent with the PREPARE II trial design, this resulted in a potential reduction of 41% admissions and 36% visits to the outpatient clinic. Consequently, the initial 50% rate of hospitalisation /outpatient care could potentially diminish to 35%. As such, for the present study’s sample size analysis, we assumed that the sFlt-1/PlGF ratio including a telemonitoring strategy leads to a de-escalation of care by cumulatively reducing the frequency of pre-eclampsia-related hospital admissions and/or outpatient visits in the first week from 50% in the control group to 35% in the intervention group. Considering a loss to follow-up of 5% a sample size of approximately 470 women is required with 235 women per arm. This sample size, calculated with an α-level of 5%, will provide 90% power to detect a statistically significant difference for the primary outcome.

### Recruitment

Participants will be recruited at the obstetric outpatient clinic or obstetrical ward at one of the six participating hospitals. A standardised moment was implemented in the outpatient clinic before 20 weeks of gestation where all women receive a letter with general information on pre-eclampsia and its symptoms, including an explanation about the potential possibility to participate in this study. Women who present with suspected pre-eclampsia between 20 and 37 weeks of gestation and meet the inclusion criteria will be informed about the study by the treating clinician or a member of the study team. Recruitment began on 13 October 2025 and is expected to be completed by 31 December 2028.

### Patient and public involvement

Patient participation is integral to this study through close collaboration with the Dutch patient association for (pre-)eclampsia and haemolysis, elevated liver enzymes, low platelets (HELLP) syndrome (Hellp Foundation). The Hellp Foundation is an independent patient organisation, which provides peer support, provides information for (ex-)PE/HELLP- patients and represents women and their partners who have experienced gestational hypertension, (pre-)eclampsia or HELLP syndrome in scientific research and guidelines. The Hellp Foundation will play a crucial role by offering support and advice in the preparation, execution, and implementation phases. As a dedicated partner throughout the entire process, they will participate in progress meetings. Additionally, the Hellp Foundation will assist in disseminating research findings to (ex-)patients through their magazine, social media, and their website, ensuring effective communication and engagement with the patient community.

### Data collection

Data will be collected in a web-based registry, Castor (CDMS 2025 2.3.0.) by the principal investigator. Intended procedures related to data handling are stipulated in the data management plan before the start of the study. The computer randomly assigns a unique numeric code for every subject that bears no relation to initials or date of birth. Data handling is performed with coded data, with the key (code to personal information linkage) only available to the investigator. Persons who have access to the data include investigators, research staff, monitoring and quality assurance personnel and the Data Safety Monitoring Board (DSMB). Data will be preserved for the duration of 15 years. The handling of personal data complies with the General Data Protection Regulation.

### Statistical analysis plan

All analyses will follow the intention-to-treat principle, whereby participants are analysed in the group to which they were randomised, regardless of adherence. In addition, a per-protocol sensitivity analysis will be performed, including only participants in the intervention group for whom the sFlt-1/PlGF ratio result was available and acted on within 24 hours, and whose subsequent management adhered to the protocol-defined strategy.

The primary analysis will compare pre-eclampsia-related healthcare utilisation between study arms in the first week after baseline using a chi-square test for proportions. Secondary analyses will include incidence of pre-eclampsia and composite maternal and perinatal adverse outcomes. An economic evaluation will be performed from a societal perspective with a time horizon from inclusion to 6 weeks postpartum. Observed cost differences will be related to diagnostic yield (true positives) in a cost-effectiveness analysis, and to quality of life in a cost-utility analysis. Quality of life will be assessed biweekly using the EQ-5D-5L, with utilities derived from the Dutch tariff and quality-adjusted life years (QALYs) calculated using the area-under-the-curve method.^19^ Healthcare use will be obtained from hospital and midwifery records, and productivity losses will be measured using the adapted iPCQ. Standard Dutch reference prices and the friction cost method will be used for valuation.^20^ Missing data will be handled by multiple imputation. Differences in mean costs and effects between strategies will be estimated using bootstrapping. In a net-benefit analysis, costs will be related to QALYs and summarised in a cost-effectiveness acceptability curve. This curve plots, for each willingness-to-pay threshold per QALY gained, the probability that an intervention yields a higher net monetary benefit than the comparator. The range of thresholds will include the values typically used in the Netherlands (€20 000–€80 000 per QALY)*,* allowing decision-makers to assess cost-effectiveness within the nationally accepted WTP framework.^21 22^ A sensitivity analysis will be conducted to assess the robustness of the findings to variations in key parameters, assumptions and methodological choices. The full Statistical Analysis Plan (SAP) has been submitted as an online supplemental document and will guide all prespecified analyses.

### Data monitoring and safety

Monitoring will be performed in compliance with Good Clinical Practice and other rules and regulations to achieve high-quality research and secure patient safety. Monitoring in all sites will be executed by (internal) monitors of the LUMC according to the monitor plan.

Despite the excellent test characteristics, there remains a small chance (<5%) that women will develop pre-eclampsia or complications at home. Therefore, in accordance with the SoC, all women in the intervention arm who will return to routine antenatal care (ie, low risk) will be instructed to contact the hospital or midwife if they have any new or persisting symptoms in order to minimise the risk of complications. All adverse events reported spontaneously by participants or observed by the investigators will be recorded. An independent DSMB is established to safeguard participant safety and to monitor the overall conduct of the trial. Meetings will be scheduled at least every 6 months, with additional ad hoc meetings if emerging safety concerns or feasibility issues arise. The DSMB will review cumulative safety data, including serious adverse events (SAEs), feasibility parameters and interim analyses. A first safety review will be conducted after data from approximately 100 participants have been collected, and additional ad hoc reviews will be triggered after every 20 SAEs. A protocol-defined interim analysis will be performed after the inclusion of approximately 50% of participants to evaluate safety, feasibility and pre-eclampsia-related healthcare utilisation. The DSMB will be authorised to recommend early termination of the study in case of a statistically significant increase in SAEs, clear evidence of benefit or harm of the intervention or relevant external evidence. No formal futility analysis will be conducted, as the absence of a statistically significant effect will be considered meaningful given the societal and implementation-driven objectives of the trial.

### Ethics and dissemination

The study will be conducted according to the principles of the Declaration of Helsinki and in accordance with the Medical Research Involving Human Subjects Act (WMO) and other guidelines, regulations and Acts. Ethical approval was obtained from the Medical Ethics Committee of Leiden University Medical Centre (METC LDD) on 21 July 2025 (reference NL-009295). Participants will receive written and verbal information in either Dutch or English and will be given sufficient time to review the study details. Written informed consent will be obtained before any study procedures. Participation is voluntary and can be withdrawn at any time without any consequences for their medical care. The treating physician or study investigator may also withdraw a participant from the study for urgent medical reasons. Participants who withdraw will remain in their allocated arm for the intention-to-treat analysis.

The sponsor will submit a summary of the progress of the trial to the accredited Medical Ethical Committee (MEC) once a year. Information will be provided on the date of inclusion of the first subject, numbers of subjects included and numbers of subjects that have completed the trial, SAEs, other problems and amendments. If any amendments will be made to the original study protocol the MEC will be notified. The sponsor will also notify the accredited MEC at the end of the study within a period of 8 weeks. The end of the study is defined as the last patient’s last visit. The sponsor will notify the MEC immediately of a temporary halt of the study, including the reason of such an action. In case the study is ended prematurely, the sponsor will notify the accredited MEC within 15 days, including the reasons for the premature termination. Within 1 year after the end of the study, the sponsor will submit a final study report with the results of the study including any publications/abstracts of the study to the accredited MEC. After completing the trial and data analysis, the results of the trial will be offered for publication to an international peer-reviewed journal with the aim to submit within 1 year after completing the trial.

The full trial protocol (version 11.0, dated 27 May 2025) and the SAP (version 2.0, dated 17 September 2025) are available as online supplemental material and can be accessed via the *BMJ Open* online publication.

## Supplementary material

10.1136/bmjopen-2025-113516 online supplemental file 1

10.1136/bmjopen-2025-113516 online supplemental file 2

10.1136/bmjopen-2025-113516 online supplemental file 3
