# Supplementary material for: Cost-effectiveness of the sFlt-1/PlGF ratio and telemonitoring in managing suspected pre-eclampsia: protocol for the PREPARE II randomised controlled trial
Source: BMJ Open. 2026 Jul 2;16(7):e113516. doi: 10.1136/bmjopen-2025-113516 (PMC13331053; doi:10.1136/bmjopen-2025-113516)
Supplement: online supplemental file 1 [file bmjopen-16-7-s001.pdf]

RESEARCH PROTOCOL PREPARE II

PREdiction of Pre-eclampsia and AdveRse Events

PROTOCOL TITLE: 'PREPARE II: PREdiction of Pre-eclampsia and AdveRse Events'

**Protocol ID**	NL88527.058.24
**Short title**	PREPARE II
**EudraCT number**	Not applicable
**Version**	11
**Date**	27-05-2025
**Coordinating investigator/project leader**	[redacted]
**Principal investigator(s)**	Dr. M. Sueters<br>Dept. Obstetrics<br>Leiden University Medical Center<br>P.O. Box 9600, 2300 RC Leiden<br>E: m.sueters@lumc.nl, T: 071-5262896
**Sponsor**	Leiden University Medical Center<br>P.O. Box 9600, 2300 RC Leiden
**Subsidising party**	ZonMw "Grant number: 10390032310044"
**Independent expert (s)**	[redacted]
**Laboratory sites**	[redacted]
**Pharmacy**	Not applicable

PROTOCOL SIGNATURE SHEET

Name	Signature	Date
Sponsor/Head of Department: [redacted]	[redacted]	27-05-2025
[redacted]	[redacted]	27-05-2025
[redacted]	[redacted]	27-05-2025
[redacted]	[redacted]	27-05-2025

TABLE OF CONTENTS

1. INTRODUCTION AND RATIONALE 9
2. OBJECTIVES 10
   - 2.1 Objective 10
   - 2.2 Hypothesis 10
   - 2.3 Research question 10
3. STUDY DESIGN AND PLAN 11
   - 3.1 Study design 11
   - 3.2 Recruitment procedure 12
   - 3.3 Study duration and follow-up per participant 12
4. STUDY POPULATION 13
   - 4.1 Population (base) 13
   - 4.2 Inclusion criteria 13
   - 4.3 Exclusion criteria 13
   - 4.4 Sample size calculation 14
5. TREATMENT OF SUBJECTS 15
   - 5.1 Investigational intervention 15
   - 5.2 Comparison/SoC 15
   - 5.3 Use of co-intervention 15
6. METHODS 17
   - 6.1 Study parameters/endpoints 17
   - 6.2 Randomization, blinding and treatment allocation 17
   - 6.3 Study procedures 18
   - 6.4 Additional study related procedures 18
   - 6.5 Withdrawal of individual subjects 20
   - 6.6 Specific criteria for withdrawal (if applicable) 20
   - 6.7 Replacement of individual subjects after withdrawal 20
   - 6.8 Follow-up of subjects withdrawn from treatment 20
   - 6.9 Premature termination of the study 20
7. SAFETY REPORTING 21
   - 7.1 Data Safety Monitoring Board (DSMB) 21
   - 7.2 Temporary halt for reasons of subject safety 21
   - 7.3 AEs, SAEs and SUSARs 21
   - 7.4 Suspected unexpected serious adverse reactions (SUSARs) 22
   - 7.5 Annual safety report 22
   - 7.6 Follow-up of adverse events 23
8. STATISTICAL ANALYSIS 23
   - 8.1 Primary study parameter(s) 23
   - 8.2 Secondary study parameter(s) 23
   - 8.3 Interim analysis 24
9. ETHICAL CONSIDERATIONS 24
   - 9.1 Regulation statement 24

9.2 Recruitment and consent 25
9.3 Objection by minors or incapacitated subjects (if applicable) 25
9.4 Benefits and risks assessment, group relatedness 25
9.5 Compensation for injury 26
9.6 Incentives (if applicable) 26
10. ADMINISTRATIVE ASPECTS, MONITORING AND PUBLICATION 27
10.1 Handling and storage of data and documents 27
10.2 Biobank and long-term sample storage 27
10.3 Monitoring and Quality Assurance 27
10.4 Data Safety Monitoring Board (DSMB) 28
10.5 Amendments 28
10.6 Annual progress report 29
10.7 Temporary halt and (prematurely) end of study report 29
10.8 Public disclosure and publication policy 29
11. REFERENCES 30
12. APPENDICES 31

LIST OF ABBREVIATIONS AND RELEVANT DEFINITIONS

**ABR**	**General Assessment and Registration form (ABR form), the application form that is required for submission to the accredited Ethics Committee; in Dutch: Algemeen Beoordelings- en Registratieformulier (ABR-formulier)**
**AE**	**Adverse Event**
**AR**	**Adverse Reaction**
**CA**	**Competent Authority**
**CCMO**	**Central Committee on Research Involving Human Subjects; in Dutch: Centrale Commissie Mensgebonden Onderzoek**
**CV**	**Curriculum Vitae**
**DSMB**	**Data Safety Monitoring Board**
**FGR**	**Fetal Growth Restriction**
**GCP**	**Good Clinical Practice**
**GDPR**	**General Data Protection Regulation; in Dutch: Algemene Verordening Gegevensbescherming (AVG)**
**IB**	**Investigator's Brochure**
**IC**	**Informed Consent**
**METC**	**Medical research ethics committee (MREC); in Dutch: medisch-ethische toetsingscommissie (METC)**
**PIGF**	**Placental growth factor**
**(S)AE**	**(Serious) Adverse Event**
**sFlt-1**	**Soluble FMS-like tyrosine kinase 1**
**Sponsor**	**The sponsor is the party that commissions the organisation or performance of the research, for example a pharmaceutical<br>company, academic hospital, scientific organisation or investigator. A party that provides funding for a study but does not commission it is not regarded as the sponsor, but referred to as a subsidising party.**
**WMO**	**Medical Research Involving Human Subjects Act; in Dutch: Wet Medisch-wetenschappelijk Onderzoek met Mensen**

SUMMARY

Rationale: Pre-eclampsia remains a leading cause of morbidity and mortality worldwide for both mother and child, complicating 2-8% of pregnancies. The sFlt-1/PlGF ratio has demonstrated impressive predictive test characteristics in women with suspected pre-eclampsia. However, it remains matter of debate whether the introduction of this novel test can indeed translate to a reduction in pre-eclampsia-related hospital admissions, outpatient visits and can consequently lower overall healthcare costs. The PREPARE II study aims to investigate whether the sFlt-1/PlGF ratio, along with digital self-monitoring, can reduce pre-eclampsia-related healthcare utilization in the first week following the test for women with suspected pre-eclampsia.

Objective: Primary objective: to assess whether risk-stratification using the spot urine protein-to-creatinine ratio (PCr) + sFlt-1/PlGF ratio including a telemonitoring strategy, reduces pre-eclampsia-related healthcare utilization (defined as pre-eclampsia driven admissions and outpatient visits) during the first week after testing in women with suspected pre-eclampsia.

Secondary objective: to investigate if the introduction of the PCr + sFlt-1/PlGF ratio including a tele-monitoring strategy for women with suspected preeclampsia leads to a favourable cost-effectiveness ratio. Secondary outcomes will be assessed by the investigator up to 6 weeks postpartum.

Study design: Randomized controlled trial including a cost-effectiveness analysis across six centres: Leiden University Medical Centre (LUMC), Groene Hart Hospital, Haaglanden Medical Centre, Haga Hospital, Reinier de Graaf Hospital, and Alrijne Hospital.

Study population: This study will enroll 470 women (≥16 years old) between 20 to 37 weeks of gestation with suspected pre-eclampsia due to one or more identified symptoms.

Intervention: The intervention is an algorithm based on the urine protein/creatinine ratio (PCr) + sFlt-1/PlGF ratio, along with a telemonitoring strategy. The algorithm incorporates a PCr cut-off of 30 (mg/mmol) and a sFlt-1/PlGF ratio cut-off of 38 for risk classification. Subsequent clinical follow-up recommendations are stratified based on this classification: low risk entails no additional follow-up, returning to routine antenatal care; intermediate risk includes telemonitoring; high risk necessitates immediate admission.

Main study parameters/endpoints: The primary outcome is the occurrence of pre-eclampsia-related healthcare utilization in first week after testing. Secondary outcomes are maternal/perinatal adverse events, total healthcare usage, pre-eclampsia diagnosis, quality of life, and productivity losses. A cost-effectiveness analysis from a societal perspective will be performed.

Nature and extent of the burden and risks associated with participation, benefit and group relatedness: Control arm of RCT: Standard-of-Care (SoC). Intervention arm of RCT: the physician will be recommended follow-up according to study protocol (no additional follow-up, telemonitoring, or immediate admission). After the first week, decision of the need for follow-up will be made based on treating physician' opinion following SoC. If necessary, the treating physicians can always deviate from study protocol based on their own expert opinion. Women in both groups will be asked to fill out questionnaires at different time-points.

Despite the excellent test characteristics there remains a small chance (<5%) that women will develop pre-eclampsia at home. Therefore, in accordance with the SoC, all women in the intervention arm who will return to routine antenatal care (i.e. low risk) will be instructed to contact the hospital or midwife if they have any new or persisting symptoms in order to minimize the risk of complications. All adverse events reported spontaneously by participants or observed by the investigators will be recorded.

1. INTRODUCTION AND RATIONALE

Pre-eclampsia remains a significant global health concern, affecting 3-5% of pregnancies and posing risks to both mothers and infants.(1-4) Pre-eclampsia is a syndrome or collection of symptoms to identify pregnant women at risk of developing serious, and even life-threatening complications such as seizures, intracranial haemorrhage, pulmonary oedema, hepatic hematoma/rupture, acute kidney injury, coagulopathy, placental abruption, fetal growth restriction and fetal death. Therefore, women presenting with symptoms leading to suspicion of pre-eclampsia often require hospitalization and/or intensive monitoring.(2, 5)

However, the current diagnostic procedures in standard care which involve blood pressure measurements and urine protein-to-creatinine ratio (PCr), inadequately identify pregnant women at risk of developing pre-eclampsia and associated complications.(6, 7) As a result, women with symptoms linked to pre-eclampsia may undergo unnecessary hospitalization and intensive monitoring until pre-eclampsia is ruled out. These admissions, along with unforeseen additional visits to the outpatient clinic in addition to routine antenatal visits, impose a substantial burden on pregnant women and contribute to potentially avoidable high healthcare costs.(8, 9)

Recent studies exploring soluble FMS-like tyrosine kinase-1 (sFlt-1) and placental growth factor (PlGF) levels in pregnant women suggest potential value in predicting the absence of pre-eclampsia.(10, 11) The PROGNOSIS study demonstrated that a sFlt-1/PlGF ratio of <38 had a negative predictive value of 99.3% for ruling out development of pre-eclampsia within the next week.(10) Ruling out the development of pre-eclampsia for a certain time period in women suspected for the disease may lead to a reduction in over-diagnosis, redundant admission, over-treatment and will consequently lower the costs.(11) Notwithstanding the impressive test characteristics, studies like INSPIRE, PARROT-UK/Ireland, have shown minimal changes in hospital admissions and complication rates, emphasizing that the efficacy of this novel test in a real-world setting remains debatable.(12-14)

In the PREPARE I study, the sFlt-1/PlGF ratio was investigated in the setting of daily clinical care in the Netherlands which includes routine testing for urine PCr in women with suspected pre-eclampsia.(15) This study demonstrated the sFlt-1/PlGF ratio in addition to PCr, may lead to improved selection of women at risk and a reduction of hospital care without compromising safety of mothers and neonates. This raises the hypothesis that a more refined selection of women at risk for complications should be accompanied by a corresponding de-escalation of care.

Therefore, we have designed the PREPARE II multicentre randomized controlled trial investigating the PCr + sFlt-1/PlGF ratio including a telemonitoring strategy for women with suspected pre-eclampsia.

2. OBJECTIVES

2.1 Objective

Primary objective: to assess whether risk-stratification using the PCr + sFlt-1/PlGF ratio including a telemonitoring strategy, reduces pre-eclampsia-related healthcare utilization (defined as pre-eclampsia driven admissions and outpatient visits) during the first week after testing in women with suspected pre-eclampsia.

Secondary objective: to investigate if the introduction of the PCr + sFlt-1/PlGF ratio including a tele-monitoring strategy for women with suspected preeclampsia leads to a favourable cost-effectiveness ratio.

2.2 Hypothesis

The intervention based on the PCr + sFlt-1/PlGF ratio including telemonitoring of the women at intermediate risk allows the de-escalation of care with a reduction of pre-eclampsia-related healthcare utilization in women with suspected pre-eclampsia without difference in maternal/perinatal adverse outcomes, improving QALY's and decreasing productivity losses from paid an unpaid work, leading to a favourable cost-effectiveness ratio.

2.3 Research question

Does the PCr + sFlt-1/PlGF ratio including a telemonitoring strategy lead to a de-escalation of care by reducing pre-eclampsia-related healthcare utilization in women with suspected pre-eclampsia compared to Standard-of-Care (SoC) without difference in maternal/perinatal adverse outcomes?

3. STUDY DESIGN AND PLAN

3.1 Study design

Multicenter randomized controlled trial including a cost-effectiveness analysis across six centers: LUMC, Groene Hart Hospital, Haaglanden Medical Centre, Haga Hospital, Reinier de Graaf Hospital, and Alrijne Hospital. Randomization will be performed on patient level. Start study is planned for the 1st of April 2025. We expect to reach the aimed sample size within 4 years. A schematic overview of the study design is shown in Figure I.

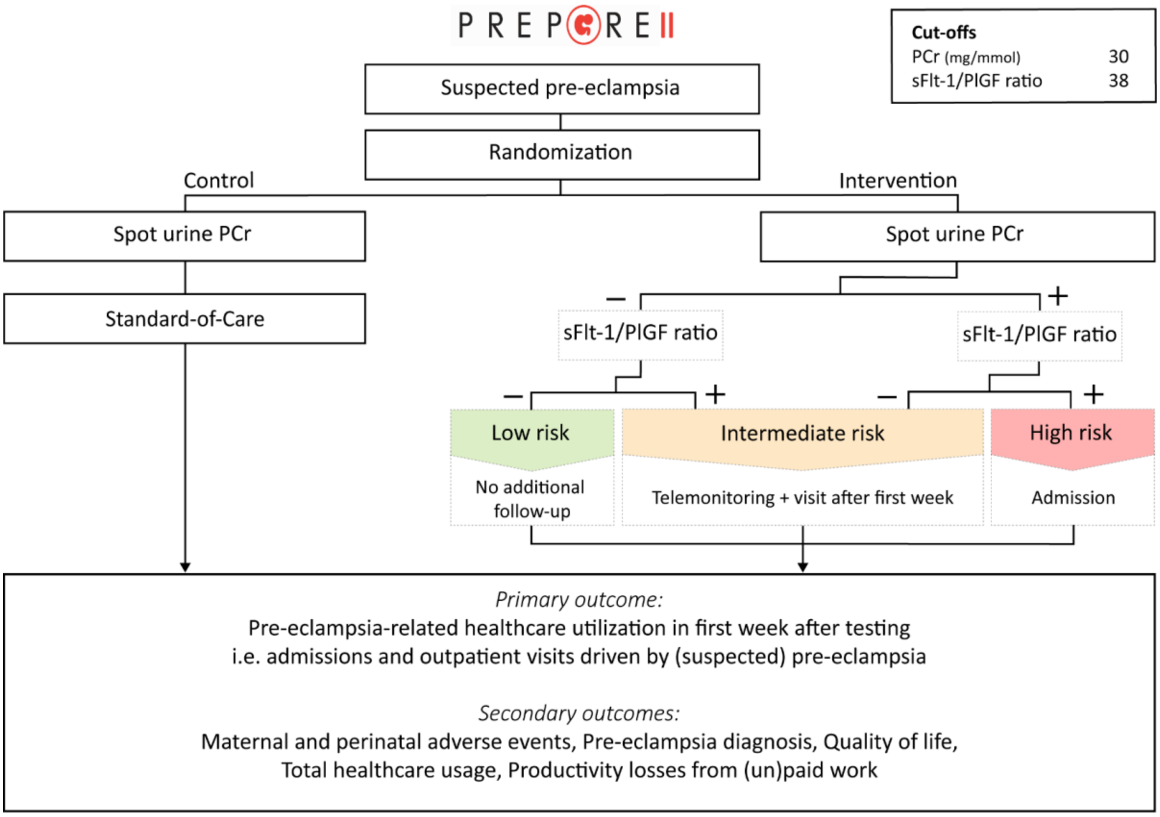

Figure I. Study design PREPARE II

PCr = Protein/Creatinine ratio, sFlt-1/PlGF = Soluble FMS like tyrosine kinase 1/Placenta Growth Factor. Women with suspected pre-eclampsia will be randomized at first presentation in the control group following SoC or the intervention arm. Follow-up in the intervention arm will be determined for each group based on this flow chart: low risk: no additional follow-up; intermediate risk: telemonitoring + weekly visit; high risk: direct admission.

3.2 Recruitment procedure

Women presenting at the outpatient clinic, emergency department or obstetrical ward with suspected pre-eclampsia will be identified and screened for eligibility by the treating physician or member of the research team. Eligibility will be assessed using the predefined inclusion and exclusion criteria.

If eligible, patients will receive verbal and written information about the study from the investigator or clinical staff. If the patient expresses interest, informed consent will be discussed and obtained. The participant herself will sign the informed consent form, confirming voluntary participation.

To ensure timely start of study procedures, informed consent must be obtained within 1 hour after presentation. This limited time window is necessary to allow real-time decision-making and implementation of follow-up based on biomarker results, particularly for those randomized to the intervention arm.

After informed consent is obtained, the participant will be randomized through Castor EDC to either the control or intervention arm. In both groups, a blood sample will be collected immediately after randomization. However, sFlt-1/PlGF analysis will only be performed in participants allocated to the intervention arm. In the control group, the blood sample will be stored but not analyzed during the study.

All participants will be asked to complete study-related questionnaires (including quality of life and productivity loss) at baseline and throughout the follow-up period until six weeks postpartum.

3.3 Study duration and follow-up per participant

For each participant, study participation starts at the moment of inclusion following the eligibility check and signed informed consent. After inclusion and randomization, blood samples will be taken for measurement of the sFlt-1/PlGF ratio and PCr. These samples will be processed immediately to determine the appropriate follow-up strategy, depending on the allocation to either the intervention or control arm.

- In the intervention arm, follow-up will start as soon as the test result is available (within 24 hours), based on the risk classification (low, intermediate, high).
- In the control arm, participants will receive follow-up according to standard clinical practice (Standard-of-Care) without knowledge of the test results.

Participants in both groups will be followed until 6 weeks postpartum, during which clinical outcomes will be assessed through chart review and questionnaires. Questionnaires regarding quality of life and productivity loss (EQ-5D-5L and iPCQ) will be completed at baseline and then every two weeks until delivery, with a final follow-up at 6 weeks postpartum.

The total study duration per participant is therefore approximately:

- From inclusion to delivery (variable, depending on gestational age at inclusion),
- Plus 6 weeks postpartum follow-up.

4. STUDY POPULATION

4.1 Population (base)

Pregnant women with suspected pre-eclampsia.

4.2 Inclusion criteria

Subjects enrolled in the study must meet all of the following criteria:

1. Age ≥ 16 years
2. Singleton pregnancy
3. Gestational age from 20 weeks + 0 days to 36 weeks + 6 days
4. Clinical suspicion of pre-eclampsia, defined as at least **one** of the following (adapted from ISSHP 2021 and NVOG 2019 guidelines):

   a. New-onset hypertension:
   - Systolic blood pressure ≥140 mmHg and/or
   - Diastolic blood pressure ≥90 mmHg
     (measured using validated equipment and standard clinical procedures.

   b. Worsening of pre-existing hypertension:
   - Increase of ≥10 mmHg systolic and/or diastolic compared to baseline, or
   - Increased need for antihypertensive medication,
     as assessed by the treating physician based on clinical context

   c. New-onset proteinuria, defined as:
   - Proteinuria detected by dipstick ≥2+
     Note: The protein/creatinine ratio (PCR) is not part of the initial inclusion criteria and will be assessed after inclusion for risk stratification and care planning.

   d. Worsening of known proteinuria, defined as:
   - Increase of ≥50% in protein-to-creatinine ratio compared to previous measurement
     Note: This applies to patients with known proteinuria (e.g., from chronic kidney disease), where it's unclear if the increase is due to their underlying condition or superimposed pre-eclampsia.

   e. Other clinical signs or symptoms suggestive of pre-eclampsia, such as:
   - Epigastric pain (especially right upper quadrant)
   - Severe oedema or sudden swelling of face, hands, or feet
   - Headache or visual disturbances (e.g., blurred vision, scotomas)
   - Sudden weight gain >1 kg/week (in third trimester)
   - Thrombocytopenia (platelet count <150 × 10^9/L)
   - Elevated liver enzymes (ALT >41 U/L or AST >40 U/L)
   - (Suspected) fetal growth restriction (FGR) based on clinical or ultrasound findings

4.3 Exclusion criteria

Subjects are excluded from participation if they meet any of the following exclusion criteria:

1) Confirmed pre-eclampsia at time of enrolment

Note: Women with a confirmed diagnosis of pre-eclampsia prior to presentation are excluded. Those with initial symptoms or findings suggestive of pre-eclampsia, but without a formal diagnosis, are eligible. A diagnosis made after randomization is considered part of the clinical course and a study outcome.

2) Any sFlt-1/PlGF ratio measured this pregnancy
3) Previous participation in the PREPARE II study in current or prior pregnancy
4) Gestational age ≥37 weeks
5) Multiple pregnancy
6) Inability to give informed consent

4.4 Sample size calculation

Using the primary outcome of pre-eclampsia-related healthcare utilization in the first week after testing, we based our assumptions on the previously conducted PREPARE I study.(15) In this study in SoC, 50% of the women with suspected pre-eclampsia were either admitted, seen at the outpatient clinic or assigned to home monitoring in the first week after baseline. In test scenario 2, consistent with the PREPARE II trial design, this resulted in a potential reduction of 41% admissions and 36% visits to the outpatient clinic. Consequently, the initial 50% rate of hospitalization/outpatient care could potentially diminish to 35%. As such, for the present study's sample size analysis, we assumed that the sFlt-1/PlGF ratio including a telemonitoring strategy leads to a de-escalation of care by cumulatively reducing the frequency of preeclampsia-related hospital admissions and/or outpatient visits in the first week from 50% in the control group to 35% in the intervention group. Considering a loss to follow-up of 5% a sample size of approximately 470 women is required with 235 women per arm. This sample size, calculated with an α-level of 5%, will provide 90% power to detect a statistically significant difference for the primary outcome.

5. TREATMENT OF SUBJECTS

5.1 Investigational intervention

In the intervention arm of the RCT, risk classification is based on PCr and sFlt-1/PlGF ratio.

Risk classification:

- Low = PCr <30 and sFlt-1/PlGF ratio ≤38
- Intermediate = PCr <30 and sFlt-1/PlGF ratio >38 or PCr ≥30 and sFlt-1/PlGF ratio ≤38
- High = PCr ≥30 and sFlt-1/PlGF ratio >3

Clinical follow-up recommendations are based on risk classification:

- Low = no additional follow-up/return to routine antenatal care
- Intermediate = telemonitoring + visit to outpatient clinic after first week
- High = direct admission

Note: clear indications for admission, as documented in local protocols (such as severe hypertension or fetal growth restriction), should never be nullified by study procedures. If deemed necessary, the healthcare provider may deviate from the research protocol based on their own expertise or the patient's presentation.

5.2 Comparison/SoC

Women with suspected pre-eclampsia in the control arm will receive standard care (SoC) follow-up at the discretion of the treating physician, which may include outpatient clinic visits or hospital admission. Standard care is provided in accordance with the Dutch national guideline on hypertensive disorders in pregnancy (NVOG, 2019). Clinical evaluation includes blood pressure measurement, urine protein/creatinine ratio (PCr), and laboratory testing, with decisions guided by the ISSHP 2021 criteria. Follow-up is determined based on clinical and laboratory findings. The sFlt-1/PlGF ratio is not revealed to the clinician in the control arm.

5.3 Use of co-intervention

The most important co-intervention of this study is telemonitoring, which is a digital platform enabling home blood pressure measurements and pre-eclampsia symptoms reporting, in line with the SAFE@HOME study.(16) For telemonitoring, pregnant women are instructed to perform blood pressure measurements and report symptoms (headache, visual symptoms, epigastric pain, swelling etc). Daily reviews of participant-uploaded results will be performed by the investigator. The data platform will generate automated alerts if blood pressure readings exceed 140/90 mmHg or if responses in the app raise concerns about pre-eclampsia. These alerts will be communicated to the responsible obstetrician, who will then discuss management options and provide further instructions to the women at home. If there is a substantial suspicion of pre-eclampsia, patients will be promptly referred to the outpatient clinic for evaluation.

Following the initial week of telemonitoring, the decision to continue this form of monitoring will be at the discretion of the treating physician. Should it be deemed necessary, treating physicians are permitted to deviate from the study protocol, based on their professional judgment. Such deviations from the protocol are not considered part of the primary or secondary endpoints of the study. However, they will be documented and reported to evaluate the percentage of adherence to the study protocol.
To assess quality of life and productivity losses from paid/unpaid work, structural surveys will be conducted electronically which include a 10 minute questionnaire (EQ-5D-5L and iPCQ). These surveys will be repeated every two weeks until delivery.

6. METHODS

6.1 Study parameters/endpoints

Main study parameter/endpoint

Pre-eclampsia-related healthcare utilization in first week after testing, defined as:

- Pre-eclampsia-related admissions defined as admissions driven by suspected pre-eclampsia or pre-eclampsia in differential diagnosis as documented by the physician in a structural questionnaire when a patient is admitted.
- Pre-eclampsia-related outpatient visits defined as a visit to the outpatient clinic driven by suspected pre-eclampsia or pre-eclampsia in differential diagnosis in addition to the routine antenatal visits as documented by the physician.

Secondary study parameters/endpoints

- Actual development of Pre-eclampsia according to the classification criteria of the ISSHP 2018 definition (2)
- Composite of maternal adverse outcomes
    - The occurrence of death, stroke, eclampsia, blindness, hypertension requiring administration of intravenous antihypertensives, the use of inotropic agents, thromboembolic events (arterial, venous or small vessel thrombosis, other than superficial venous thrombosis, in any tissue or organ), pulmonary oedema (diagnosed clinically with one/more of oxygen saturation <95%, diuretic treatment or x-ray confirmation), respiratory failure (needing intubation), myocardial ischemia or infarction, hepatic dysfunction (leading to disseminated intravascular coagulation), hepatic hematoma or rupture (confirmed by imaging or at laparotomy), renal failure (serum creatinine >200 µmol/L), and transfusion of any blood products. One patient can have more than one adverse outcome. We will report on the frequency of pregnant women encountering at least one adverse outcome.
- Composite of perinatal adverse outcomes
    - Preterm delivery (spontaneous and iatrogenic before 37 and 32 weeks), fetal growth restriction (FGR, birthweight <10th percentile), admission to the neonatal intensive care-unit (NICU) and perinatal death. One newborn can have more than one adverse outcome. We will report on the frequency of newborns encountering at least one adverse outcome.
- Change in quality of life (EQ-5D-5L and anxiety/stress)
- Total healthcare usage (i.e. admissions, home-monitoring, telemonitoring and outpatient visits beyond first week after baseline)
- Productivity losses from paid and unpaid work (iPCQ).

6.2 Randomization, blinding and treatment allocation

Women will be randomized in a 1:1 ratio to either the intervention or control arm at the time of inclusion. The local researcher conducting the inclusion will not have prior knowledge of the patient's allocation to a trial arm. The randomization process will be facilitated by Castor EDC, an electronic data capture system. Randomization will occur at the individual patient level stratified for centre of inclusion and gestational age below 34 weeks or 34 weeks and later. Randomization will occur blinded at the laboratory level independent of the treating

physician. The following interventions, as detailed below, will and cannot be blinded due to the nature of the intervention.

6.3 Study procedures

Women presenting with suspected pre-eclampsia between 20 and 37 weeks of gestation at the obstetrical ward or outpatient clinic at one of the study sites will be invited to participate. Following informed consent, the clinician will randomize the participant into either the control group (receiving SoC with no knowledge of the sFlt-1/PlGF ratio result) or the intervention group (utilizing the PCr + sFlt-1/PlGF ratio algorithm) using Castor EDC. Subsequently, the clinician will document the trial arm allocation and the rationale for inclusion in the electronic health record. Additionally, 20 mL of blood will be collected through venipuncture from both control and intervention groups, and, if taken in the secondary care participating centers, sent to the clinical chemistry laboratory in Leiden via lab transport. Samples from the intervention group will be transported via emergency/private transport, while samples from the control group will be sent via regular transport.

Upon availability of the test result, the treating clinician will be notified by the clinical chemistry department only if the participant is allocated to the intervention arm. Throughout the period until the test result is obtained (with a maximum duration until next morning), participants will adhere to SoC regardless of their study arm allocation. In the intervention arm, the sFlt-1/PlGF ratio result will be disclosed and a proposed protocolised follow-up plan for the patient is given, being one of the following: no additional follow-up, telemonitoring, or hospital admission. It is crucial to emphasize that the sFlt-1/PlGF ratio result does not influence decisions regarding induction of labour or medication administration.

If the participant is assigned to the control arm, the test result remains undisclosed, and they will continue to follow SoC based on the treating physician's discretion. The results of the sFlt-1/PlGF ratio in the control arm will be revealed to the investigators upon completion of the study for a pre-defined post-hoc analysis modelling the flow and healthcare usage of patients according to the unrevealed test result. A schematic overview of the schedule of enrolment is provided in Table I.

6.4 Additional study related procedures

Biobank

The additional blood sample collected for the PREPARE Biobank will be used for future research into the pathophysiology, prediction, and prevention of pre-eclampsia and related maternal and perinatal complications.

The rationale for long-term biobank storage (up to 20 years) is:

- Scientific value: Pre-eclampsia is a heterogeneous and complex disorder with unclear underlying mechanisms. Long-term access to well-characterized samples from a prospective cohort allows future biomarker, genomic, and immunologic studies as new hypotheses and technologies emerge.
- Rarity and timing: Pre-eclampsia occurs in a subset of pregnancies and has unpredictable onset. Collecting biological samples at first presentation is logistically

and ethically only feasible during ongoing clinical studies. Without prospective collection, future studies would be limited or biased.
- Infrastructure: The LUMC houses a certified biobank facility with secure, regulated long-term storage. Samples will only be used in future ethically approved studies. Participants give separate informed consent for biobanking, and are free to decline without any impact on participation in the PREPARE II trial.

Questionnaires

All women will be asked to fill out questionnaires. When informed consent and email address is provided, patients will receive an email with a personal link to the questionnaires they can fill out online. At baseline a short questionnaire (EQ-5D-5L and iPCQ) will be filled out to assess quality of life and productivity losses from paid/unpaid work. This questionnaire will be repeated every 2 weeks till delivery.

		**Study Period**				
**Timepoints**	Presentation at outpatient clinic with suspected PE	Baseline and randomization	First week after baseline	Every 2 weeks till delivery	Delivery (in hospital or at home)	Close out visit 6 weeks postpartum
Enrolment						
Eligibility screen	X					
Informed consent	X					
Allocation		X				
Interventions (in-person)						
sFlt-1/PlGF measurement		X				
Follow-up decision after 24h		X				
No follow-up/telemonitoring/ Admission			X			
Questionnaire (EQ5D, iPCQ)		X		X		
Assessments (charts)						
Demographics		X				
History, comorbidities		X				
Physical measurements		X				
Clinical readings		X				
Maternal outcomes					X	X
Perinatal outcomes					X	X
Prenatal hospital admissions						X
Outpatient visits						X

Table I. Schedule of enrolment, interventions, and assessments in PREPARE II study

6.5 Withdrawal of individual subjects

Subjects can leave the study at any time for any reason if they wish to do so without any consequences. The investigator can decide to withdraw a subject from the study for urgent medical reasons. Women who withdraw from the study will remain in their treatment group for the intent-to-treat analysis, their data will be used for analysis. Every effort will be made to obtain complete information on each patient randomized. The only reason for not obtaining complete information is that the patient was lost to follow-up, incomplete questionnaires or that she withdraws consent to access her medical chart after delivery. Once a participant has been randomized, even though she refuses the study intervention for any reason, follow-up will be continued including the planned visits, maternal and fetal surveillance, which are part of SoC.

6.6 Specific criteria for withdrawal (if applicable)

Not applicable

6.7 Replacement of individual subjects after withdrawal

Not applicable

6.8 Follow-up of subjects withdrawn from treatment

Not applicable

6.9 Premature termination of the study

The study may be terminated prematurely following a recommendation by the Data Safety Monitoring Board (DSMB). As outlined in the DSMB charter, such a recommendation will be based on expert clinical judgment and a comprehensive assessment of participant safety and trial feasibility. No formal statistical stopping boundaries have been predefined.

<u>Stopping criteria</u>

The DSMB will give highest priority to participant safety. The committee may recommend early termination of the trial under the following conditions:

- A clinically relevant safety concern, such as an increased frequency or severity of serious adverse events (SAEs), as judged by the DSMB;
- A clear and clinically relevant difference in the primary outcome is observed, and further inclusion is unlikely to add value for interpretation or future implementation.

Early termination will not be based on feasibility parameters alone (e.g., recruitment rate, protocol adherence, or site logistics), and no formal futility analysis will be conducted. The absence of a statistically significant effect is considered a meaningful outcome in itself, particularly given the societal and implementation-focused aim of the study (ZonMw-funded).
Further details on DSMB procedures and decision-making processes are provided in the DSMB charter (Appendix A).

SAFETY REPORTING

6.10 Data Safety Monitoring Board (DSMB)

An independent Data Safety Monitoring Board (DSMB) has been established for the PREPARE II study to monitor participant safety and study integrity. The DSMB is responsible for the periodic evaluation of safety data, including serious adverse events (SAEs), and for providing recommendations based on interim evaluations. These evaluations may address both safety and feasibility aspects of the trial. The structure, responsibilities, and procedures of the DSMB are outlined in the DSMB charter, included as Appendix A of this protocol.

The DSMB will convene at least once annually and may meet more frequently if required based on accumulating safety data. A formal interim safety review will be conducted after 100 participants have completed follow-up, and SAE summaries will be provided after every 20 reported SAEs. An additional interim feasibility evaluation may be scheduled when approximately 235 participants have been enrolled.

All DSMB recommendations will be communicated to the sponsor. If the sponsor decides not to fully implement a DSMB recommendation, the rationale for this decision will be documented and shared with the reviewing Medical Ethics Review Committee (METC). In such cases, other relevant regulatory authorities will also be informed where applicable.

6.11 Temporary halt for reasons of subject safety

In accordance to section 10, subsection 4, of the WMO, the sponsor will suspend the study if there is sufficient ground that continuation of the study will jeopardise subject health or safety. The sponsor will notify the accredited METC without undue delay of a temporary halt including the reason for such an action. The study will be suspended pending a further positive decision by the accredited METC. The investigator will take care that all subjects are keptinformed.

6.12 AEs, SAEs and SUSARs

Adverse events (AEs)

Adverse events are defined as any undesirable experience occurring to a subject during the study, whether or not considered related to the experimental intervention. All adverse events of grade 3 or more (Common Terminology Criteria for Adverse Events (CTCAE) v5.0: severe or medically significant but not immediately life-threatening; hospitalization or prolongation of hospitalization indicated) reported spontaneously by the participant or observed by the investigator or his staff will be recorded.

Serious adverse events (SAEs)

A serious adverse event is any untoward medical occurrence or effect that

- results in maternal or fetal death;
- is life threatening (at the time of the event) to the mother or the fetus;

- requires hospitalisation or prolongation of existing inpatients' hospitalisation;
- results in persistent or significant disability or incapacity;
- any other important medical event that did not result in any of the outcomes listed above due to medical or surgical intervention but could have been based upon appropriate judgement by the investigator.

The investigator will report all SAEs to the sponsor without undue delay after obtaining knowledge of the events. The sponsor will report the SAEs through the web portal *ToetsingOnline* to the accredited METC that approved the protocol, within 7 days of first knowledge for SAEs that result in death or are life threatening followed by a period of maximum of 8 days to complete the initial preliminary report. All other SAEs will be reported within a period of maximum 15 days after the sponsor has first knowledge of the serious adverse events.

Maternal hospital admission driven by suspected pre-eclampsia or pre-eclampsia in (differential) diagnosis without severe morbidity (e.g. eclampsia, renal failure, lung oedema) is an expected complication due to the higher risk of the study population. This complication is included in the primary and secondary outcomes of this study and will be recorded in the Case Report Form. Immediate and individual reporting of this SAE will not enhance the safety of the study. Therefore, this SAE does not need to be reported individually through *ToetsingOnline* like other SAEs.

The following events are *not* considered to be a Serious Adverse Event:
- Elective maternal hospital admissions
- Hospital admissions for (induction of) labour or scheduled delivery/caesarean section
- Neonatal hospital admission for observation which is not at the NICU
- Hospitalization for a procedure that was planned prior to study participation.
- Prolonged hospitalization for technical, practical, or social reasons, in absence of an adverse event.

All women in the intervention arm who will not be directly admitted (i.e. low and intermediate risk) will be instructed to contact the hospital if they have any new or persisting symptoms in order to minimize the risk of complications. As mentioned above, physicians can always deviate from study protocol based on their expert' opinion and are always allowed to admit a patient.

6.13 Suspected unexpected serious adverse reactions (SUSARs)
Not applicable; no investigational medicinal product will be used in this trial

6.14 Annual safety report
Not applicable; no investigational medicinal product will be used in this trial

6.15 Follow-up of adverse events

All SAEs will be followed until they have abated, or until a stable situation has been reached. Depending on the event, follow up may require additional tests or medical procedures as indicated, and/or referral to the general physician or a medical specialist. SAEs need to be reported till end of study within the Netherlands, as defined in the protocol.

7. STATISTICAL ANALYSIS

A statistical analysis plan will be made shortly after the start of the study.

7.1 Primary study parameter(s)

All analyses will be performed according to the intention-to-treat principle. The primary analysis will compare the pre-eclampsia related health care usage among the two study arms in the first week after baseline using a chi-square test for proportions. Secondary, we compare total hospital admissions, outpatient visits, pre-eclampsia diagnosis and composite outcome of maternal/perinatal adverse events between the groups. Outcomes will be adjusted for gestational age at inclusion (per stratification in the randomization) using a linear regression model. If women with suspected pre-eclampsia allocated to the intervention arm will be less often admitted to the hospital without comprising safety in the first week after baseline, we will conclude that follow-up including sFlt-1/PlGF ratio and telemonitoring could be beneficial.

7.2 Secondary study parameter(s)

Cost-effectiveness analysis

An economic evaluation will be performed from a societal perspective comparing the diagnostic yield (i.e. true positives) in a cost-effectiveness analysis and to differences in quality of life in a cost-utility analysis. Because sFlt-1/PlGF ratio testing is new in the Dutch healthcare system and used by this study, we anticipate a pre-defined sensitivity study conducting a per-protocol analysis of the primary and all secondary endpoints. As such, the per-protocol analysis of the study can investigate the subset of patients for whom the physician truly had the timely (i.e. within 24 hours or within the same day) availability of sFlt-1/PlGF ratio test outcome during the study. The cost-effectiveness analysis will be performed from a societal perspective with a time horizon from inclusion till 6 weeks postpartum. Due to the short time horizon, costs and effects will not be discounted. Quality of life will be measured by means of the EQ-5D-5L every 2 weeks from inclusion till 6 weeks postpartum. Utilities will be calculated from the EQ-5D-5L questionnaire using the Dutch tariff.(17) Using the area-under-the-curve method, QALYs can be obtained from the utility measurements over time. Healthcare use will be obtained from hospital and midwifery registrations and absence from work will be measured by an adapted version of iPCQ tested in the pilot study (including pregnancy related productivity losses), healthcare use will be valued using standard intervention with SoC. The observed differences in costs will be related to the costs.(18) Absence from work will be valued with friction cost method.

Budget impact analysis

Implementation scenarios will be evaluated from the relevant perspectives (societal, healthcare, healthcare insurance), in accordance with the Dutch BIA guidelines.

7.3 Interim analysis

An interim evaluation will be conducted after approximately 235 participants have been enrolled, representing around 50% of the total target sample size. This evaluation will focus on the feasibility and safety of the study procedures, including an assessment of pre-eclampsia-related healthcare utilization during the first week after presentation in both study arms.

This evaluation is not a formal futility analysis, and no predefined statistical stopping boundaries have been set. The aim is to support the DSMB in assessing the ongoing practical feasibility, participant safety, and operational implementation of the trial. The absence of a statistically significant effect will not be considered a reason to terminate the study prematurely.

The evaluation will be performed by an independent statistician, and results will be submitted to the DSMB in blinded format, with the option for unblinding at the DSMB's discretion.

In addition to this evaluation, the DSMB will conduct a separate interim safety review after 100 participants have completed full follow-up. Summaries of Serious Adverse Events (SAEs) will also be provided after every 20 reported SAEs.

Based on its assessment, the DSMB may advise to:

- Continue the trial as planned;
- Recommend modifications to sample size or study procedures;
- Or, in the case of a clinically relevant safety concern or a clear difference in the primary outcome, advise early termination if further inclusion is deemed unlikely to add interpretive or implementation value.

DSMB recommendations will be based on cumulative safety data, trial conduct, and expert clinical judgment.

Further information regarding the responsibilities, scope, and procedures of the DSMB is available in section 6.10 and the DSMB charter (Appendix A).

8. ETHICAL CONSIDERATIONS

8.1 Regulation statement

The study will be conducted according to the principles of the Declaration of Helsinki (see for the most recent version: www.wma.net) and in accordance with the Medical Research Involving Human Subjects Act (WMO) and other guidelines, regulations and Acts.

8.2 Recruitment and consent

We implemented a standardized moment in the outpatient clinic before 20 weeks of gestation where all women receive a letter with general information on pre-eclampsia and its symptoms, including explanation about the potential possibility to participate in this study. Women with suspected pre-eclampsia who present at the obstetrical ward or the outpatient clinic and meet the inclusion criteria are informed about the study by treating clinician and the investigator. Women are given sufficient time to read the information and informed consent form in English or Dutch. If a patient agrees to participate in the study, written informed consent is obtained for participation in the study. Each subject must be informed that participation in the study is voluntary, and that withdrawal of consent will not affect her right to the most appropriate medical treatment or affect the doctor relationship. Women can contact the researchers by telephone for further information and to ask questions.

8.3 Objection by minors or incapacitated subjects (if applicable)

Not applicable

8.4 Benefits and risks assessment, group relatedness

Women participating in the control arm of the RCT do not incur additional risks compared to women not participating in this study as they will receive follow-up according to SoC.
The additional study-related procedures women undergo in the control arm (blood sampling, fill out questionnaires) do not oppose additional risks and the burden of these procedures are considered minimal.

Women in the intervention arm of the RCT will receive follow-up as recommended per protocol based on the PCr and sFlt-1/PlGF result: no additional follow-up, telemonitoring, or hospital admission. In previously conducted studies the negative predictive value of the sFlt-1/PlGF ratio ruling out the development of pre-eclampsia was high, which implies this test can be safely implemented. (10, 12, 15) Furthermore, the SAFE@HOME study has proven that a clinical pathway with telemonitoring for women at risk of pre-eclampsia allows fewer antenatal visits, ultrasounds and hypertension-related admissions, with no differences in perinatal outcomes. (16, 19) Women in the intervention arm are therefore likely to have a benefit, as they may be less often admitted and may have to visit the hospital less frequently compared to women in the control arm.

However, despite the excellent test characteristics there remains a small chance (<10%) that women will develop pre-eclampsia or complications at home. Therefore, all women in the intervention arm who will not be directly admitted (i.e. low and intermediate risk) will be instructed to contact the hospital if they have any new or persisting symptoms in order to minimize the risk. As mentioned above, physicians can always deviate from study protocol based on their expert' opinion and are always allowed to admit a patient.

8.5 Compensation for injury

The sponsor/investigator has a liability insurance which is in accordance with article 7 of the WMO.

The sponsor (also) has an insurance which is in accordance with the legal requirements in the Netherlands (Article 7 WMO). This insurance provides cover for damage to research subjects through injury or death caused by the study.
The insurance applies to the damage that becomes apparent during the study or within 4 years after the end of the study.

8.6 Incentives (if applicable)

Not applicable

9. ADMINISTRATIVE ASPECTS, MONITORING AND PUBLICATION

9.1 Handling and storage of data and documents

Data will be collected in a web-based registry (Castor EDC) by the principal investigator. Upon inclusion, the system will automatically assign a unique numeric code to each participant. This code bears no relation to initials or date of birth and is used for all study-related documentation and data handling.

All data will be stored in coded format. The key to this code — which links the numeric study ID to the participant's personal identifiers — is stored securely and separately at the coordinating center (LUMC). This key is only accessible to the principal investigator and designated study staff when necessary.

Persons who may access coded data include the investigators, research staff, monitors, quality assurance personnel, and members of the DSMB. Direct access to identifiable personal data is restricted to authorized staff only and solely for the purpose of study-related monitoring, data verification, or regulatory review.

Data will be preserved for a period of 15 years after study completion. The handling of personal data is fully compliant with the General Data Protection Regulation (GDPR) (in Dutch: Algemene Verordening Gegevensbescherming, AVG).

9.2 Biobank and long-term sample storage

If the participant has provided separate informed consent for biobanking, an additional blood sample will be collected at the time of study inclusion. This sample will be stored for future research related to pre-eclampsia and reproductive immunology.

Samples from participating secondary care centres will be temporarily stored locally and transported in batches to the LUMC for long-term storage. At the LUMC, these samples will be stored in the PREPARE Biobank under controlled and secure conditions.

Biobank storage will be for a maximum of 20 years, in accordance with Dutch laws and regulations, and only accessible for future ethically approved studies. If participants do not consent to biobank storage, all remaining material will be destroyed after initial analysis.

9.3 Monitoring and Quality Assurance

Monitoring will be performed in compliance with Good Clinical Practice (GCP), the WMO, and other applicable regulatory requirements to ensure high-quality research and the protection of participant safety.

Monitoring will be carried out at all participating sites in the Netherlands by (internal) monitors from the LUMC. These monitors will operate according to a pre-defined monitoring plan, which outlines the frequency, scope, and nature of monitoring visits (e.g. initiation, interim, and close-out visits).

During these visits, monitors will:

- Verify informed consent procedures;
- Check data entered into the electronic Case Report Forms (eCRFs) against source documents;
- Ensure adherence to the study protocol and applicable regulations;
- Review safety reporting and adverse event documentation;
- Confirm proper storage and security of study-related documents and data.

Findings and deviations will be documented in monitoring reports and communicated to the study team for corrective actions when necessary. If substantial issues are identified, the principal investigator and sponsor will be notified, and follow-up actions will be initiated.

All monitoring activities will be logged and archived in accordance with GCP standards.

9.4 Data Safety Monitoring Board (DSMB)

An independent Data Safety Monitoring Board (DSMB) has been established to perform ongoing safety surveillance throughout the PREPARE II study. The DSMB is responsible for monitoring participant safety, including Serious Adverse Events (SAEs), and may advise on continuation, modification, or early termination of the study in the event of **clinically relevant** safety concerns or clear outcome-related findings, as outlined in the DSMB charter.

The DSMB will meet at least once annually, with additional meetings scheduled:

- After 100 participants have completed full follow-up (interim safety review),
- After approximately 235 participants have been enrolled (feasibility and safety evaluation),
- And after every 20 reported SAEs (ad hoc review).

A DSMB charter detailing its responsibilities, procedures, and composition is included as Appendix A to this protocol.

The advice of the DSMB will be communicated to the sponsor. If the sponsor decides not to (fully) implement the DSMB's recommendations, the sponsor will forward the recommendation with written justification to the reviewing METC. Relevant health authorities will be informed if applicable.

All DSMB activities and recommendations will be documented and retained in the Trial Master File.

9.5 Amendments

Amendments are changes made to the research after a favourable opinion by the accredited METC has been given. All amendments will be notified to the METC that gave a favourable opinion.

9.6 Annual progress report

The sponsor/investigator will submit a summary of the progress of the trial to the accredited METC once a year. Information will be provided on the date of inclusion of the first subject, numbers of subjects included and numbers of subjects that have completed the trial, serious adverse events/ serious adverse reactions, other problems, and amendments.

9.7 Temporary halt and (prematurely) end of study report

The investigator/sponsor will notify the accredited METC of the end of the study within a period of 8 weeks. The end of the study is defined as the last patient's last visit. The sponsor will notify the METC immediately of a temporary halt of the study, including the reason of such an action. In case the study is ended prematurely, the sponsor will notify the accredited METC within 15 days, including the reasons for the premature termination. Within one year after the end of the study, the investigator/sponsor will submit a final study report with the results of the study, including any publications/abstracts of the study, to the accredited METC.

9.8 Public disclosure and publication policy

After completing the trial and data analysis, the results of the trial will be published as soon as possible in an international journal on obstetrics.

10. REFERENCES

1. Magee LA, Brown MA, Hall DR, Gupte S, Hennessy A, Karumanchi SA, et al. The 2021 International Society for the Study of Hypertension in Pregnancy classification, diagnosis & management recommendations for international practice. Pregnancy Hypertens. 2022;27:148-69.
2. Brown MA, Magee LA, Kenny LC, Karumanchi SA, McCarthy FP, Saito S, et al. Hypertensive Disorders of Pregnancy: ISSHP Classification, Diagnosis, and Management Recommendations for International Practice. Hypertension. 2018;72(1):24-43.
3. Say L, Chou D, Gemmill A, Tunçalp Ö, Moller AB, Daniels J, et al. Global causes of maternal death: a WHO systematic analysis. Lancet Glob Health. 2014;2(6):e323-33.
4. Wang W, Xie X, Yuan T, Wang Y, Zhao F, Zhou Z, et al. Epidemiological trends of maternal hypertensive disorders of pregnancy at the global, regional, and national levels: a population-based study. BMC Pregnancy Childbirth. 2021;21(1):364.
5. Chappell LC, Cluver CA, Kingdom J, Tong S. Pre-eclampsia. Lancet. 2021;398(10297):341-54.
6. Zhang J, Klebanoff MA, Roberts JM. Prediction of adverse outcomes by common definitions of hypertension in pregnancy. Obstet Gynecol. 2001;97(2):261-7.
7. Hagmann H, Thadhani R, Benzing T, Karumanchi S, A., Stepan H. The promise of angiogenic markers for the early diagnosis and prediction of preeclampsia. Clin Chem. 2012;58(5):837-45.
8. Stevens W, Shih T, Incerti D, Ton TGN, Lee HC, Peneva D, et al. Short-term costs of preeclampsia to the United States health care system. Am J Obstet Gynecol. 2017;217(3):237-48.e16.
9. Delahaije DH, Smits LJ, van Kuijk SM, Peeters LL, Duvekot JJ, Ganzevoort W, et al. Care-as-usual provided to formerly preeclamptic women in the Netherlands in the next pregnancy: health care consumption, costs and maternal and child outcome. Eur J Obstet Gynecol Reprod Biol. 2014;179:240-5.
10. Zeisler H, Hund M, Verlohren S. The sFlt-1:PlGF Ratio in Women with Suspected Preeclampsia. N Engl J Med. 2016;374(18):1785-6.
11. Vatish M, Strunz-McKendry T, Hund M, Allegranza D, Wolf C, Smare C. sFlt-1/PlGF ratio test for pre-eclampsia: an economic assessment for the UK. Ultrasound Obstet Gynecol. 2016;48(6):765-71.
12. Cerdeira AS, O'Sullivan J, Ohuma EO, Harrington D, Szafranski P, Black R, et al. Randomized Interventional Study on Prediction of Preeclampsia/Eclampsia in Women With Suspected Preeclampsia: INSPIRE. Hypertension. 2019;74(4):983-90.
13. Duhig KE, Myers J, Seed PT, Sparkes J, Lowe J, Hunter RM, et al. Placental growth factor testing to assess women with suspected pre-eclampsia: a multicentre, pragmatic, stepped-wedge cluster-randomised controlled trial. Lancet. 2019;393(10183):1807-18.
14. Hayes-Ryan D, Khashan AS, Hemming K, Easter C, Devane D, Murphy DJ, et al. Placental growth factor in assessment of women with suspected pre-eclampsia to reduce maternal morbidity: a stepped wedge cluster randomised control trial (PARROT Ireland). Bmj. 2021;374:n1857.
15. Wind M, van den Akker-van Marle ME, Ballieux B, Cobbaert CM, Rabelink TJ, van Lith JMM, et al. Clinical value and cost analysis of the sFlt-1/PlGF ratio in addition to the spot urine protein/creatinine ratio in women with suspected pre-eclampsia: PREPARE cohort study. BMC Pregnancy Childbirth. 2022;22(1):910.
16. van den Heuvel JFM, Lely AT, Huisman JJ, Trappenburg JCA, Franx A, Bekker MN. SAFE@HOME: Digital health platform facilitating a new care path for women at increased risk of preeclampsia - A case-control study. Pregnancy Hypertens. 2020;22:30-6.
17. Versteegh MM, Vermeulen KM, Evers SMAA, de Wit GA, Prenger R, Stolk EA. Dutch Tariff for the Five-Level Version of EQ-5D. Value Health. 2016;19(4):343-52.
18. Hakkaart-van Roijen L, Peeters S, Kanters T. Costing manual for economic evaluations in healthcare: Methodology and Reference prices [in Dutch: Kostenhandleiding voor economische evaluaties in de gezondheidszorg: Methodologie en Referentieprijzen]. 2024.
19. van den Heuvel JFM, van Lieshout C, Franx A, Frederix G, Bekker MN. SAFE@HOME: Cost analysis of a new care pathway including a digital health platform for women at increased risk of preeclampsia. Pregnancy Hypertens. 2021;24:118-23.

11. APPENDICES

APPENDIX A: DSMB CHARTER

**1. Introduction**	
Name (and sponsor's ID) of trial<br>ISRCTN and/or EUDRACT number	PREPARE II: PREdiction of Pre-eclampsia and AdveRse Events<br>METC nummer: NL-009295<br>CTR:
Objectives of trial, including interventions being investigated	PREPARE II is a multicenter randomized controlled trial investigating whether the addition of the sFlt-1/PlGF ratio and digital telemonitoring to current standard risk stratification for suspected pre-eclampsia can reduce pre-ecampsia-related healthcare utilization. Approximately 470 women will be randomized across six hospitals in the Netherlands.
Outline of scope of charter	The purpose of this document is to describe the roles and responsibilities of the independent Data Safety and Monitoring Committee (DSMB) for the PREPARE II trial, including the timing of meetings, methods of providing information to and from the DSMB, frequency and format of meetings, statistical issues and relationships with other committees.
**2. Roles and responsibilities**	
Aims of the committee	To safeguard the interests of trial participants, assess the safety and feasibility of the trial procedures, and monitor the overall conduct of the clinical trial.
Terms of reference	The DSMB should receive and review the progress and accruing data of this trial and provide advice on the conduct of the trial.<br>The DSMB should inform the principal investigator if, in their view:<br>(i) The results are likely to convince a broad range of clinicians that one trial arm is clearly indicated or contraindicated, and there is a reasonable expectation that this new evidence would materially influence patient management; or<br>(ii) A statistically significant difference in the primary outcome is observed at interim, and further inclusion is unlikely to provide added value for interpretation or implementation.<br><br>For operational reasons, the DSMB will inform the clinical trials coordination team of its decisions. In case of material recommendations (e.g. when suspension or termination of the study is recommended), these will also be communicated to the sponsor's representative (head of department).
General roles of the DSMB	• monitor evidence for treatment harm<br>• decide whether to recommend that the trial continues to recruit participants or whether recruitment should be terminated either for everyone or for some treatment groups and/or some participant

	subgroups<br>• assess the impact and relevance of external evidence
**3. Before or early in the trial**	
Any issues specific to the disease under study	All DSMB members will have insight into the protocol before agreeing to monitor the trial. The DSMB members are specifically asked to comment on the proposed method of DSMB oversight and interim analyses planned.
Whether the DSMB will meet before the start of the trial	The DSMB will have insight in the study protocol and may suggest protocol changes before start of the trial. Within six months after start of the trial the study will be presented to an open DSMB meeting.
Any other issues specific to the treatment under study	The study evaluates a diagnostic and triage strategy based on biomarker-guided risk classification and home blood pressure monitoring (telemonitoring). Although no therapeutic intervention is tested, the DSMB should consider the implications of this approach in a vulnerable pregnant population, including patient safety and operational feasibility.
Any specific regulatory issues	None
Any other issues specific to the treatment under study	None
Whether members of the DSMB will have a contract	There will be no contract. The DSMB members will sign a ‘no conflict of interest statement’, on this form they also state to fully agree with this DSMB charter.
**4. Composition**	
Membership and size of the DSMB	The members of the DSMB for this trial are:<br>[redacted]

	[redacted]
The responsibilities of the DSMB statistician	The statistician of the committee, [redacted] will attend all meetings; she will provide statistical advice and recommendations. The interim analysis report will be produced [redacted] who will submit the report directly to the DSMB for review and discussion.
The responsibilities of the PI and other members of the Trial Project Group (TPG)	The principal investigator (dr. M. Sueters) and coordinating researcher only provide input to the production of the non-confidential sections of the DSMB report.<br>The project leader should be available to attend open sessions of the DSMB meeting where indicated. The other TPG members will not usually be expected to attend but may attend open sessions when necessary (See Organisation of DSMB Meetings).

5. Relationships

Relationships	Relationships The DSMB plays an advisory role. If the advice of the DSMB is neglected or overruled, the medical ethical committee will be informed
Clarification of whether the DSMB are advisory (make recommendations) or executive (make decisions)	The DSMB plays an advisory role.
Payments to DSMB members	DSMB members will be reimbursed for travel expenses only.
The need for DSMB members to disclose information about any competing interests	All competing interests will be disclosed at the first DSMB meeting, and will be updated in case of material changes.

6. Organisation of DSMB meetings

Expected frequency of DSMB meetings	The DSMB will meet at predefined intervals and on an ad hoc basis to monitor participant safety, review trial conduct, and ensure data integrity. The planned DSMB meeting schedule is as follows:<br>• **Regular Meetings**<br>The DSMB will convene at least every six months to assess cumulative safety data, monitor recruitment, and review protocol adherence.<br>• **Planned Safety Review**<br>A dedicated safety review will be held after outcome data from approximately 100 participants are available. This review is intended to identify any early safety concerns.

	• **Ad Hoc Meetings**<br>The DSMB will meet on an ad hoc basis if 20 or more Serious Adverse Events (SAEs) are reported, or if unexpected safety signals emerge. In such cases, the DSMB will receive a comprehensive line listing of all SAEs.<br>• **Feasibility Assessment**<br>An additional review focused on trial feasibility and recruitment progress will be conducted once data are available for approximately 235 participants.<br>• **Final Meeting**<br>A concluding DSMB meeting may be held at the end of the trial to assess overall safety data prior to study close-out.
Whether meetings will be face-to-face or by teleconference	In principle the meetings will be face-to-face. Additional teleconference meetings can be held if indicated.
How DSMB meetings will be organised, especially regarding open and closed sessions, including who will be present in each session	The DSMB meetings will be structured into an open and a closed session. The initial meeting will begin with an open session, during which the coordinating researcher will present the study background and current progress. This session will be attended by the principal investigator.<br><br>Subsequently, a closed session will be held exclusively for DSMB members. Interim data assessments and relevant trial updates will be reviewed during these closed sessions. The DSMB statistician will interpret the interim analysis findings. If necessary, the principal investigator may be invited to provide clarification, but will not be present during deliberations or decision-making.
**7. Trial documentation and procedures to ensure confidentiality and proper communication**	
Intended content of material to be available in open sessions	Open session:<br>The protocol will be explained and clarified in the first session.<br>Accumulating information relating to recruitment and data quality (e.g. data return rates, treatment compliance) will be presented. Total numbers of events for the primary outcome measure and other outcome measures may be presented, at the discretion of the DSMB.
Intended content of material to be available in closed sessions	Closed sessions:<br>In addition to all the material available in the open session, the closed session material will include safety data by treatment group as described in the protocol.
Will the DSMB be blinded to the treatment allocation	The DSMB will be blinded, but may be unblinded by the DSMB statistician on request without further justification. This request will be granted in all cases.<br>In general, the DSMB will most likely assess the unblinded data before arriving at a material recommendation.

Who will see the accumulating data and interim analysis	The accumulating data on safety and feasibility will be reviewed exclusively by the DSMB statistician and all members of the DSMB. The study team will only have access to aggregate information on the number of (serious) adverse events, without access to treatment allocation or outcome-specific data.<br><br>The DSMB members are strictly bound to confidentiality and are not permitted to share any unblinded or sensitive information with individuals outside the DSMB, including the principal investigator or other members of the study team.
Who will be responsible for identifying and circulating external evidence (e.g. from other trials/ systematic reviews)	The principal investigator will be responsible for identifying and circulating external evidence.
To whom the DSMB will communicate the decisions/ recommendations that are reached	Decisions or recommendations that are reached will be communicated to the principal investigator of the study dr. M. Sueters. When results of an interim analysis have been reviewed, a copy of the DSMB's recommendations will also be sent to the independent statistician who conducted the analysis ([redacted]). In case of material recommendations (e.g. suspension or termination of the study), these will also be communicated to the sponsor's representative (head of department). To ensure timely decisions, this will be done within 3 weeks after the DSMB meeting.
Whether reports to the DSMB be available before the meeting or only at/during the meeting	Prior to the first meeting the protocol and other relevant material related to the trial will be sent to the DSMB; this is planned to be sent at least 3-4 weeks before the meeting.<br><br>Interim analysis data will be sent to the Chair and secretary of the DSMB directly by the DSMB statistician. The Chair may decide whether a face-to-face meeting is required, or the decision can be made by a telephone conference.
What will happen to the confidential documents after the meeting	The DSMB members should store the documents safely after each meeting so they may check the next report against them. After the trial is reported, the DSMB members should destroy all interim reports.<br><br>The DSMB statistician will ensure that after the trial is completed, interim analysis data is archived securely within the institute of the principal investigator or study coordinator.

8. Decision making

What decisions/recommendations will be open to the DSMB	<u>Early stopping may be recommended in case of:</u><br>• A statistically significant increase in Serious Adverse Events (SAEs) in the intervention group;<br>• Clinically relevant safety concerns, even without statistical significance;<br>• A statistically significant difference in the primary outcome at interim analysis, and if further inclusion is unlikely to provide added value for interpretation or implementation.

	No formal futility analysis will be conducted.<br>The absence of a statistically significant effect is still considered a meaningful outcome. Early stopping for futility is not deemed appropriate given the study's societal and implementation-driven objectives (ZonMw funding), and would risk losing clinically relevant insights.
The role of formal statistical methods, specifically which methods will be used and whether they will be used as guidelines or rules	For this trial, we plan a protocol-defined interim analysis for the primary endpoint (pre-eclampsia-related healthcare utilization within 1 week after presentation) when outcome data are available for approximately 235 participants, representing around 50% of the total sample size.<br>To support the DSMB's general roles, further non-inferential interim analyses on safety and feasibility will be performed at predefined points during the trial:<br>• A first interim analysis focused on safety will take place after outcome data are available for the first 100 participants.<br>• Additional ad hoc safety reviews will be triggered after every 20 Serious Adverse Events (SAEs) reported.<br>The DSMB may advise to stop the trial at any moment when the safety of the patients is considered to be in danger. These reasons should be noted. When the principal investigator deviates from the advice given by the DSMB to stop the trial, the principal investigator will inform the METC of the divergent views.
How decisions or recommendations will be reached within the DSMB	Every effort should be made for the DSMB to reach a unanimous decision. If the DSMB cannot achieve this, a vote may be taken, although details of the vote should not be routinely included in the report to the investigators as these may inappropriately convey information about the state of the trial data.
When the DSMB is quorate for decision-making	The DSMB will consist of four members. The committee is quorate for decision-making if at least three members including the statistician are attending the meeting. Effort should be made for all members to attend. The trials office team will try to ensure that a date is chosen to enable this, therefore 4 face-to-face meetings are planned in advance in January of each year.<br>If, at short notice, any DSMB members cannot attend then the DSMB may still meet if at least one statistician and one clinician, including the Chair (unless otherwise agreed) will be present. If the DSMB is considering recommending major action after such a meeting the DSMB Chair should inform the absent members as soon after the meeting as possible to check they agree. If they do not, a teleconference should be arranged with the full DSMB.
Can DSMB members who cannot attend the meeting input	If the report is circulated before the meeting, DSMB members who will not be able to attend the meeting may pass comments to the DSMB Chair for consideration during the discussions.
What happens to members who do not attend meetings	If a member does not attend a meeting, it should be ensured that the member is available for the next meeting. If a member does not attend a second meeting, they should be asked if they wish to remain part of the DSMB.<br>If a member does not attend a third meeting, (s)he may be replaced if

	considered necessary for the proper functioning of the DSMB.
Whether different weight will be given to different endpoints (e.g. safety/efficacy)	**Safety**<br>The safety of participants will be given highest priority. Specific stopping guidelines for safety are formulated in the protocol. The DSMB may recommend early termination of the trial if:<br><br>• There is a statistically significant increase in Serious Adverse Events (SAEs) in the intervention group compared to the control group ($p < 0.05$); or<br>• A concerning pattern of harm is identified based on clinical judgment, even in the absence of statistical significance.<br><br>To support this, the DSMB will receive cumulative safety data alongside blinded comparative summaries. Safety assessments will take place:<br><br>• After outcome data are available for the first 100 participants (scheduled safety review)<br>• And after every 20 reported SAEs (ad hoc reviews)<br><br>**Feasibility**<br>In addition to safety, the DSMB will monitor feasibility parameters such as recruitment rate, protocol adherence, and site performance. However, feasibility concerns alone will not lead to early trial termination.<br><br>The only scenario in which feasibility may justify early stopping is:<br><br>• If a statistically significant difference in the primary outcome is observed during the interim analysis and if further inclusion is unlikely to provide added value for the interpretation or implementation of results.<br><br>**Futility**<br>No formal futility analysis will be conducted. The absence of a statistically significant effect is still considered a meaningful outcome. Early stopping for futility is not appropriate given the study's societal and implementation-driven objectives (ZonMw funding). Doing so would risk losing clinically relevant insights that can only emerge with full data collection.
Any specific issues relating to the trial design that might influence the proceedings, e.g. cluster trials, equivalence trials, multi-arm trials	This is a two-arm, individually randomized superiority trial. While the trial design is conventional, some specific elements may influence DSMB procedures:<br><br>• Risk-stratified follow-up: In the intervention arm, clinical follow-up is based on a biomarker-driven risk classification (low, intermediate, high). This approach may lead to variations in care pathways within the intervention group.<br>• Digital home monitoring: Participants in the intermediate-risk group receive telemonitoring. While this is non-invasive and already used in clinical practice, it may affect healthcare utilization or the timing of clinical interventions, and therefore merits monitoring.

**9. Reporting**	
To whom will the DSMB report their recommendations/decisions, and in what form	Decisions will be communicated by a formal letter within 3 weeks, with a copy to the DSMB statistician who performed the interim analysis. If no safety issues are identified, this will also be confirmed by letter. These letters will be sent by the chair of the DSMB on its behalf.
Whether minutes of the meeting be made and, if so, by whom and where they will be kept	Minutes of the open session of the meeting will be made by a trial office staff member. If (s)he cannot be present, (s)he will assign someone else to this task. The minutes will be checked by the Chair of the DSMB.<br>Minutes of the closed session of the meeting will be made by the DSMB.
What will be done if there is disagreement between the DSMB and the body to which it reports	If the DSMB has serious problems or concerns with the decision of the principal investigator, a meeting of the trial project team and the DSMB should be held.<br>The information to be shown would depend upon the action proposed and the DSMB's concerns. Depending on the reason for the disagreement confidential data will have to be revealed to all those attending such a meeting. The meeting should be chaired by a senior member of the trials unit staff or an external expert who is not directly involved with the trial.
**10. After the trial**	
Publication of the results	The trial protocol will state that results will be published in a correct and timely matter. The DSMB will not standard provide input for the publication, but they may give advice about data interpretation if they consider it necessary, or based on questions from the principal investigator.
The information about the DSMB that will be included in published trial reports	DSMB members will be named and their affiliations listed in the acknowledgements of the main report and journal publication, unless they explicitly request otherwise.<br>A brief summary of the timings and conclusions of DSMB meetings may be included in the main trial manuscript.<br>The trial project group will share with the DSMB the final report or main publication of the trial.
Any constraints on DSMB members divulging information about their deliberations after the trial has been published	The DSMB may discuss issues from their involvement in the trial 3 months after the primary trial results have been published. The DSMB will always inform the trial project group.

Appendix B: Potential conflict of interest form for Data Monitoring Committee members for the PREPARE II trial.

Principal Investigator: Dr. M. Sueters
Sponsor Name: Leiden University Medical Center (LUMC)
Funder of the Trial: ZonMw – File number: 10390032310044

This form should be completed during the instalment of the DSMB for the trial **PREPARE II**.

The avoidance of any perception that members of a DSMB may be biased in any way is important for the credibility of the decisions made by the DSMB and for the integrity of the trial.

All possible competing interests must be disclosed at the first meeting of the DSMB, when the DSMB is formally installed. If the conflict of interest is considered substantial, the member should remove the conflict or resign from the DSMB.

As potential competing interests we consider:

- Stock ownership in any commercial companies involved
- Stock transactions in any commercial company involved (if previously holding stock)
- Consulting arrangements with the sponsor
- Frequent speaking engagements on behalf of the intervention
- Career tied to a product or technique assessed by the trial
- Hands-on participation in the trial
- Involvement in the running of the trial
- Emotional involvement in the trial
- Intellectual conflict, e.g. strong prior belief in the trial's experimental arm
- Involvement in regulatory issues relevant to the trial procedures
- Investment (financial or intellectual) in competing products
- Involvement in the publication

1. Do you have any competing interest for being DSMB member for the PREPARE II trial?

☐ No, I have no competing interests to declare.
☐ Yes, I have competing interests to declare, details:
………
………
………

2. Do you agree to be a member of the DSMB for the PREPARE II trial?

☐ I have considered my potential conflicts of interest and declare to be a member of the DSMB of the PREPARE II trial, and accept the associated roles and responsibilities as described in the DSMB charter.

Name: ___________________________________

Signature: _______________________________

Date: ____________________________________
