# Supplementary material for: Cost-effectiveness of the sFlt-1/PlGF ratio and telemonitoring in managing suspected pre-eclampsia: protocol for the PREPARE II randomised controlled trial
Source: BMJ Open. 2026 Jul 2;16(7):e113516. doi: 10.1136/bmjopen-2025-113516 (PMC13331053; doi:10.1136/bmjopen-2025-113516)
Supplement: online supplemental file 2 [file bmjopen-16-7-s002.pdf]

STATISTICAL ANALYSIS PLAN (SAP) - PREPARE II study

PREdiction of Pre-eclampsia and AdveRse Events

Version: 2.0
Date: 17-09-2025
Prepared by: Aniek Peters
Study ID: NL-009295
Study Title: PREPARE II – Prediction of Pre-eclampsia and Adverse Events
Sponsor: Leiden University Medical Center
Trial Registration Number: NL88527.058.24
METC Number: NL-009295

Table of Contents

1. Introduction 3
2. Study Objectives and Endpoints 4
    2.2 Secondary Objectives 5
3. Methods and Design 6
    3.1 Study Design 6
    3.2 Eligibility Criteria 6
    3.3 Randomization 8
    3.3. Study Intervention and Follow-up 8
    3.4 Sample Size Determination 10
    3.4 Ethics approval 11
4. Statistical Analysis 11
    4.1 Trial profile 11
    4.2 General Statistical Considerations 12
    4.3 Baseline characteristics 13
    4.3 Primary Outcome Analysis 15
    4.4 Secondary Outcomes Analysis 16
        4.4.1 Maternal and Perinatal Adverse Outcomes 16
        4.4.2.Pre-eclampsia Diagnosis 19
        4.4.3. Quality of Life and Work Productivity 19
        4.4.4. Cost-Effectiveness Analysis 20
        4.4.5. Budget Impact Analysis 22
    4.5 Exploratory Analyses: Pre-eclampsia Incidence by Risk Category 23
5. Data Management and Monitoring 24
6. Interim Analysis 25
7. Discussion 26

1. Introduction

Pre-eclampsia affects 3-5% of pregnancies, posing serious risks to both mothers and infants. Due to potential complications, pregnant women with suspected pre-eclampsia often require hospitalization and intensive monitoring. (1–5) However, current diagnostic methods, including blood pressure measurements and protein-to-creatinine ratio (PCr), have limited accuracy in identifying women truly at risk. (6,7) As a result, many women experience unnecessary admissions and additional healthcare visits, increasing both patient burden and healthcare costs. (8,9)

Recent research suggests that the sFlt-1/PlGF ratio may help predict the absence of pre-eclampsia, potentially reducing unnecessary interventions. The PROGNOSIS study demonstrated that an sFlt-1/PlGF ratio <38 had a 99.3% negative predictive value for ruling out pre-eclampsia within one week. (10,11) However, studies such as INSPIRE and PARROT-UK/Ireland indicate that its real-world impact on hospital admissions remains uncertain. (12,13)

The PREPARE I study evaluated the sFlt-1/PlGF ratio alongside PCr in Dutch clinical practice, showing improved risk stratification and potential for reducing hospital care without compromising safety. (14) Building on these findings, PREPARE II is a multicentre randomized controlled trial (RCT) assessing whether a PCr + sFlt-1/PlGF ratio approach, combined with telemonitoring, can optimize risk assessment, reduce unnecessary admissions, and improve cost-effectiveness.

This Statistical Analysis Plan (SAP) outlines the analytical approach to assessing the primary and secondary endpoints of the PREPARE II study. The statistical methods were selected

based on the study design and the nature of the outcomes, ensuring a robust evaluation of pre-eclampsia-related healthcare utilization, maternal and neonatal outcomes, quality of life, and economic impact.

The analysis will follow CONSORT guidelines for RCTs, ICH-GCP standards, and established economic evaluation frameworks to ensure methodological rigor and transparency.

The intended audience for this SAP includes study investigators, statisticians, the Data Safety Monitoring Board (DSMB), and regulatory authorities, providing a clear and structured framework for data analysis, interpretation, and reporting.

2. Study Objectives and Endpoints

2.1 Primary Objective

The primary objective of the PREPARE II study is to evaluate whether the use of the sFlt-1/PlGF ratio combined with telemonitoring reduces pre-eclampsia-related healthcare utilization within one week after testing.

- **Pre-eclampsia-related hospital admissions**: Defined as hospitalizations due to suspected pre-eclampsia or pre-eclampsia as part of the differential diagnosis, as documented by the treating physician in a structured questionnaire.
- **Pre-eclampsia-related outpatient visits**: Defined as additional visits to the outpatient clinic beyond routine antenatal care due to suspected pre-eclampsia or pre-eclampsia as part of the differential diagnosis, as documented by the treating physician.

2.2 Secondary Objectives

The secondary objectives of the study are to evaluate the broader impact of the intervention beyond the primary outcome. These include the incidence of pre-eclampsia, maternal and neonatal complications, delivery outcomes, health-related quality of life, overall healthcare utilization, and the economic implications of the intervention.

- **Development of pre-eclampsia**: Diagnosed according to the ISSHP 2018 classification criteria. (2)
- **Composite of maternal adverse outcomes**: Including severe hypertension (systolic ≥ 160 mmHg and diastolic ≥ 110 mmHg), eclampsia, cerebral hemorrhage, HELLP syndrome, renal insufficiency, hematological complications, elevated liver transaminases, pulmonary edema, disseminated intravascular coagulation, pulmonary embolism, placental abruption, and maternal death.
- **Composite of perinatal adverse outcomes**: Including preterm delivery (<37 and <32 weeks), fetal growth restriction (< 10th percentile), 5-minute Apgar scores, dysmaturity, dysmaturity (< 5th percentile), asphyxia, respiratory distress syndrome requiring respiratory support, NICU admission and neonatal death.
- **Change in quality of life**: Evaluating maternal physical and psychological well-being from inclusion until six weeks postpartum.
- **Total healthcare utilization beyond the first week**: Including all hospital admissions, outpatient visits, home monitoring, and telemonitoring episodes not captured in the primary outcome.
- **Productivity losses from paid and unpaid work**: Assessing the extent of absenteeism and reduced productivity due to illness or medical intervention.

3. Methods and Design

3.1 Study Design

The PREPARE II study is a multicenter, parallel-group, randomized controlled trial (RCT) designed to assess whether the addition of sFlt-1/PlGF ratio testing in combination with telemonitoring improves risk stratification and clinical management in pregnant women with suspected pre-eclampsia.

The trial will be conducted across six hospitals in the Netherlands: Leiden University Medical Center (LUMC), Groene Hart Hospital, Haaglanden Medical Centre, Haga Hospital, Reinier de Graaf Hospital, and Alrijne Hospital.

Women between 20+0 and 37+0 weeks of gestation who present with clinical suspicion of pre-eclampsia at the obstetric ward or outpatient clinic of one of the participating centers will be eligible for inclusion. The study is scheduled to commence on 22 September 2025 and is expected to run for approximately three years, during which a total of 470 participants will be enrolled to achieve the required sample size.

3.2 Eligibility Criteria

The eligibility criteria are designed to ensure inclusion of pregnant women at risk for pre-eclampsia, while minimizing potential confounding factors.

Inclusion Criteria

Participants must meet all of the following criteria:

1. Age > 16 years

2. Singleton pregnancy

3. Gestational age from 20 weeks + 0 days to 36 weeks + 6 days

4. Clinical suspicion of pre-eclampsia, defined as at least **one** of the following (adapted from ISSHP 2021 guidelines) (4) :

   a. New-onset hypertension:

   - Systolic blood pressure ≥140 mmHg and/or
   - Diastolic blood pressure ≥90 mmHg (measured using validated equipment and standard clinical procedures.

   b. Worsening of pre-existing hypertension:

   - Increase of ≥10 mmHg systolic and/or diastolic compared to baseline, or
   - Increased need for antihypertensive medication, as assessed by the treating physician based on clinical context

   c. New-onset proteinuria, defined as:

   - Proteinuria detected by dipstick ≥2+

   d. Worsening of known proteinuria, defined as:

   - Increase of ≥50% in protein-to-creatinine ratio compared to previous measurement

   e. Other clinical signs or symptoms suggestive of pre-eclampsia, such as:

   - Epigastric pain (especially right upper quadrant)
   - Severe oedema or sudden swelling of face, hands, or feet
   - Headache or visual disturbances (e.g., blurred vision, scotomas)
   - Sudden weight gain >1 kg/week (in third trimester)
   - Thrombocytopenia (platelet count <150 × 10^9/L)
   - Elevated liver enzymes (ALT >41 U/L or AST >40 U/L)
   - (Suspected) fetal growth restriction (FGR) based on clinical or ultrasound findings

Exclusion Criteria

Participants will be excluded if they meet any of the following:

- Confirmed pre-eclampsia at the time of enrollment
- Any sFlt-1/PlGF ratio measurement performed earlier in the current pregnancy
- Previous participation in the PREPARE II study in the current or a prior pregnancy
- Gestational age < 20 weeks and ≥ 37 weeks + 0 days
- Multiple pregnancy (twins or higher-order multiples)
- Inability to provide informed consent

3.3 Randomization

Randomization is performed centrally using the Castor Electronic Data Capture (EDC) system. Participants are individually assigned in a 1:1 ratio to either the intervention or control group. Stratification is applied by gestational age (<34 weeks vs. ≥34 weeks) and participating center to ensure balanced allocation and comparability of baseline characteristics across both groups.

Participants are allocated to one of the following groups:

- Intervention group: follow-up based on risk stratification results derived from the sFlt-1/PlGF ratio in combination with PCr testing.
- Control group: Standard care alone.

Due to the nature of the intervention and the subsequent clinical management guided by group allocation, blinding of participants and treating clinicians is not feasible. Both are aware of the assigned study arm to ensure appropriate clinical follow-up.

3.3. Study Intervention and Follow-up

Participants in the intervention group will undergo a risk-based follow-up strategy informed by the combined results of the protein-to-creatinine ratio (PCr) and the sFlt-1/PlGF ratio. Risk classification is defined as follows:

- **Low risk**: PCr <30 mg/mmol and sFlt-1/PlGF ratio ≤ 38
- **Intermediate risk**: Either PCr <30 mg/mmol and sFlt-1/PlGF ratio >38, or PCr ≥ 30 mg/mmol and sFlt-1/PlGF ratio ≤ 38
- **High risk**: PCr ≥ 30 mg/mmol and sFlt-1/PlGF ratio >38

Clinical follow-up is tailored to the assigned risk category:

- Low-risk participants will return to routine antenatal care with no additional follow-up.
- Intermediate-risk participants will receive home-based telemonitoring, including remote blood pressure and symptom tracking, and a follow-up visit at the outpatient clinic after one week.
- High-risk participants will be directly admitted to the hospital for further monitoring and management.

Participants randomized to the control group will receive standard antenatal care according to national and local clinical guidelines, without access to the sFlt-1/PlGF ratio results.

Each participant is followed from enrollment until six weeks postpartum, with the total study expected to run for three years to achieve the required sample size.

A schematic overview of the study design and follow-up is presented in Figure 1.

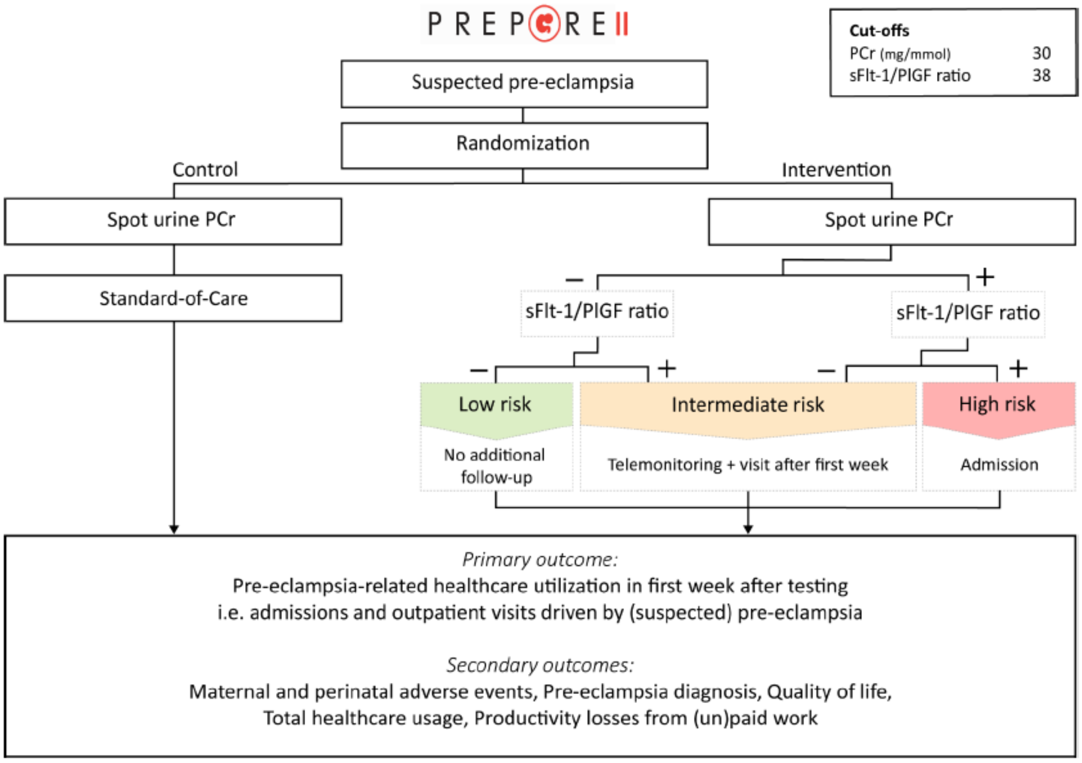

Figure 1. Study design PREPARE II

3.4 Sample Size Determination

The sample size for the PREPARE II study is calculated based on the primary outcome: pre-eclampsia-related healthcare utilization within the first week after testing. Estimates are derived from the PREPARE I study which found that approximately 50% of women with suspected pre-eclampsia in the standard care setting were either hospitalized, seen at the outpatient clinic, or received home monitoring within the first week after baseline. (14)

In a modeled scenario from PREPARE I—aligned with the PREPARE II study design—implementation of sFlt-1/PlGF ratio testing with risk-based follow-up was associated with a

41% reduction in hospital admissions and a 36% reduction in outpatient visits. This translated into a potential overall reduction of healthcare utilization from 50% to 35%.

Assuming an absolute difference of 15% between groups (50% in the control group vs. 35% in the intervention group), a total of 470 participants (235 per arm) will provide 90% power to detect a statistically significant difference, using a two-sided α-level of 0.05. The sample size includes a 5% allowance for potential loss to follow-up.

3.4 Ethics approval

The PREPARE II trial was approved by the Medical Ethics Committee of Leiden | Den Haag | Delft (METC LDD) in the Netherlands on 21 July 2025 (NL-009295), in accordance with the Dutch Medical Research Involving Human Subjects Act (WMO) and the principles outlined in the Declaration of Helsinki.

The trial was prospectively registered in the Dutch Trial Register NL88527.058.24 on 7 February 2025.

4. Statistical Analysis

4.1 Trial profile

This statistical analysis plan (version 2.0; dated 17 September 2025) is based on the PREPARE II study protocol, version 11.0, dated 27 May 2025.

The flow of participants through the trial will be illustrated using a Consolidated Standards of Reporting Trials (CONSORT) flow diagram. (15) The diagram will present the total number of participants randomized, with allocation shown for each study arm. Within each group, the

number of participants who withdrew consent, completed baseline data collection, were lost to follow-up, or contributed outcome data will be reported. An example of the participant flow is shown in Figure 2.

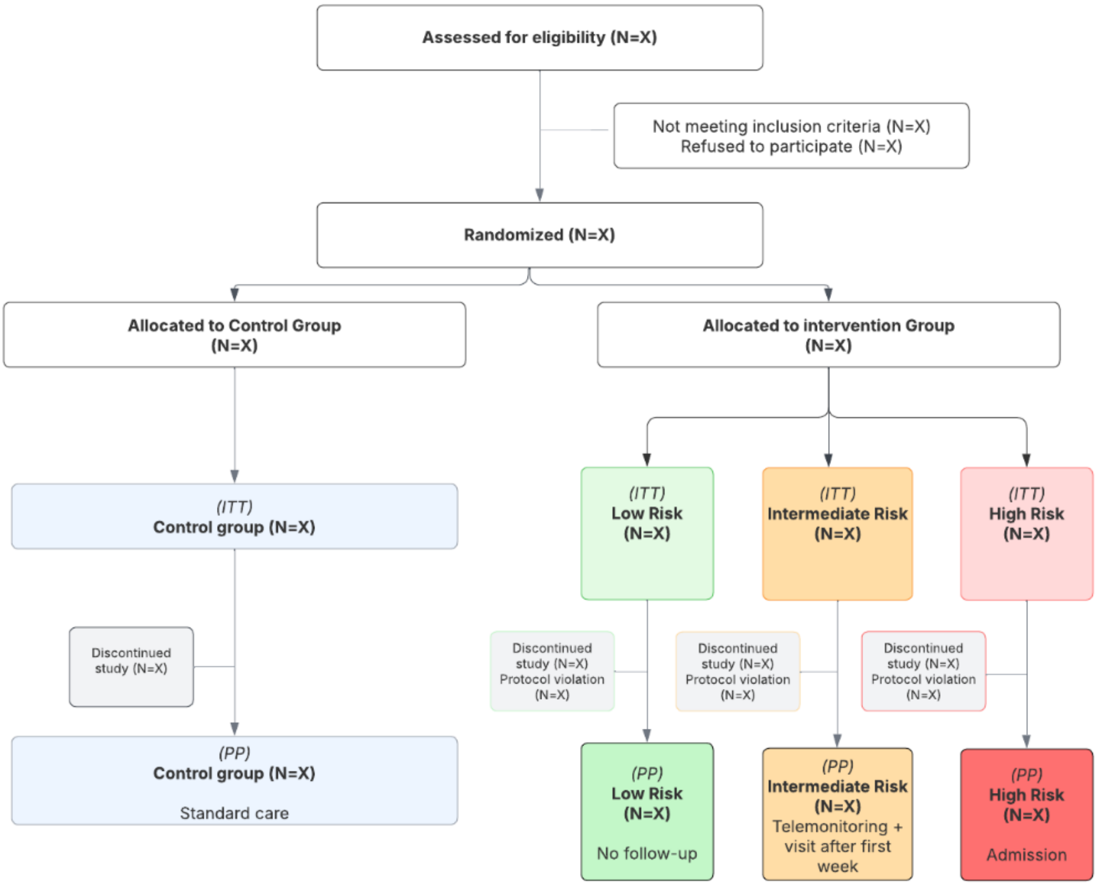

Figure 2. Flow of Participants

4.2 General Statistical Considerations

All analyses will be conducted according to the intention-to-treat (ITT) principle, whereby participants will be analyzed in the groups to which they were initially randomized, irrespective of adherence to the assigned intervention. If a participant withdraws consent

during the course of the study, only the data collected up to the time of withdrawal will be used in the final analysis.

A per-protocol (PP) sensitivity analysis will also be performed, including only participants in the intervention group for whom the sFlt-1/PlGF ratio result was available and acted upon within 24 hours after testing, and whose subsequent clinical management adhered to the protocol-defined follow-up strategy.

Missing data will be handled using multiple imputation under the assumption that data are missing at random. The number and percentage of missing observations will be reported for all relevant outcomes and covariates. Statistical tests will be two-sided, with a significance level set at $p < 0.05$.

4.3 Baseline characteristics

Baseline maternal demographic and clinical characteristics will be summarized descriptively for each randomized group. No formal statistical comparisons will be performed between groups at baseline. Categorical variables will be presented as frequencies and percentages, based on the number of non-missing observations. Continuous variables will be summarized using means and standard deviations for approximately normally distributed data, or medians and interquartile ranges (25th–75th percentile) for skewed distributions. The number of missing values will be reported for each variable. All relevant baseline characteristics will be presented in Table 1.

Patients (N)	**Total**<br>**(N=X)**	**Control group**<br>**(N=X)**	**Intervention group**<br>**(N=X)**	**Intervention group**:<br>*Low* Risk<br>**(N=X)**	**Intervention group:**<br>*Intermediate* Risk<br>**(N=X)**	**Intervention group:**<br>*High* Risk<br>**(N=X)**
**General Characteristics**						
Gestation age	Mean (SD)	Mean (SD)	Mean (SD)	Mean (SD)	Mean (SD)	Mean (SD)
Caucasian	N (X %)	N (X %)	N (X %)	N (X %)	N (X %)	N (X %)
Smoking during pregnancy	N (X %)	N (X %)	N (X %)	N (X %)	N (X %)	N (X %)
BMI (kg/m2)	Mean (SD)	Mean (SD)	Mean (SD)	Mean (SD)	Mean (SD)	Mean (SD)
**Reason of inclusion**						
New onset of hypertension	N (X %)	N (X %)	N (X %)	N (X %)	N (X %)	N (X %)
Aggravation of pre-existing hypertension	N (X %)	N (X %)	N (X %)	N (X %)	N (X %)	N (X %)
New onset of proteinuria	N (X %)	N (X %)	N (X %)	N (X %)	N (X %)	N (X %)
Epigastric pain	N (X %)	N (X %)	N (X %)	N (X %)	N (X %)	N (X %)
Excessive oedema/severe swelling	N (X %)	N (X %)	N (X %)	N (X %)	N (X %)	N (X %)
Headache	N (X %)	N (X %)	N (X %)	N (X %)	N (X %)	N (X %)
Visual disturbances	N (X %)	N (X %)	N (X %)	N (X %)	N (X %)	N (X %)
Sudden weight gain	N (X %)	N (X %)	N (X %)	N (X %)	N (X %)	N (X %)
Low platelets	N (X %)	N (X %)	N (X %)	N (X %)	N (X %)	N (X %)
Elevated liver transaminases	N (X %)	N (X %)	N (X %)	N (X %)	N (X %)	N (X %)
Fetal Growth Restriction (< 10th centile) (FGR)	N (X %)	N (X %)	N (X %)	N (X %)	N (X %)	N (X %)
**Medical History**						
Pre-existing chronic Hypertension	N (X %)	N (X %)	N (X %)	N (X %)	N (X %)	N (X %)
Autoimmune disorders (e.g. SLE)	N (X %)	N (X %)	N (X %)	N (X %)	N (X %)	N (X %)
Pre-existing cardiovascular disease	N (X %)	N (X %)	N (X %)	N (X %)	N (X %)	N (X %)
Pre-existing renal disease	N (X %)	N (X %)	N (X %)	N (X %)	N (X %)	N (X %)
Pre-existing diabetes	N (X %)	N (X %)	N (X %)	N (X %)	N (X %)	N (X %)
**Gestational Characteristics**						
Nulliparous	N (X %)	N (X %)	N (X %)	N (X %)	N (X %)	N (X %)
Gestational Diabetes Mellitus	N (X %)	N (X %)	N (X %)	N (X %)	N (X %)	N (X %)
History of pre-eclampsia	N (X %)	N (X %)	N (X %)	N (X %)	N (X %)	N (X %)
History of FGR	N (X %)	N (X %)	N (X %)	N (X %)	N (X %)	N (X %)
**Medication**						
Aspirin prophylaxis	N (X %)	N (X %)	N (X %)	N (X %)	N (X %)	N (X %)
Calcium supplement	N (X %)	N (X %)	N (X %)	N (X %)	N (X %)	N (X %)
Antihypertensive agent(s)	N (X %)	N (X %)	N (X %)	N (X %)	N (X %)	N (X %)
**Clinical characteristics**						
Mean systolic blood pressure (mmHg)	Mean (SD)	Mean (SD)	Mean (SD)	Mean (SD)	Mean (SD)	Mean (SD)
Mean Diastolic blood pressure (mmHg)	Mean (SD)	Mean (SD)	Mean (SD)	Mean (SD)	Mean (SD)	Mean (SD)
Protein/Creatinine ratio (mg/mmol) $\geq$0.3	Mean (SD)	Mean (SD)	Mean (SD)	Mean (SD)	Mean (SD)	Mean (SD)

g/24h/collected following PCr						
sFlt-1/PlGF ratio	Mean (SD)	Mean (SD)	Mean (SD)	Mean (SD)	Mean (SD)	Mean (SD)
sFlt-1 (pg/ml)	Mean (SD)	Mean (SD)	Mean (SD)	Mean (SD)	Mean (SD)	Mean (SD)
PlGF (pg/ml)	Mean (SD)	Mean (SD)	Mean (SD)	Mean (SD)	Mean (SD)	Mean (SD)

Table 1. Baseline characteristics

4.3 Primary Outcome Analysis

The primary outcome of the PREPARE II study is the proportion of participants who experience pre-eclampsia-related healthcare utilization within one week after testing. This composite outcome includes either hospital admission or an additional outpatient visit specifically related to suspected pre-eclampsia, as documented by the treating physician in a structured case report form.

The primary comparison between the intervention and control groups will be conducted using a chi-square test to evaluate differences in proportions. To further account for potential confounding, a multivariable logistic regression model will be constructed with treatment allocation as the main independent variable and pre-eclampsia-related healthcare utilization as the dependent variable.

Covariates included in the model are based on their established clinical relevance and their inclusion in validated pre-eclampsia risk prediction models. (16) The model will adjust for gestational age at inclusion (continuous), pre-existing hypertension, use of antihypertensive medication at baseline, age, body mass index (BMI), ethnicity, primigravity, and a history of severe pre-eclampsia in a previous pregnancy. Where available, additional risk factors will be included, such as maternal age, diabetes mellitus, chronic kidney disease, autoimmune disorders (e.g., systemic lupus erythematosus or antiphospholipid syndrome), conception via assisted reproductive technologies (e.g., oocyte donation), and smoking status.

		**Total**<br><br>**(N=X)**	**Control group**<br><br>**(N=X)**	**Intervention group**<br><br>**(N=X)**	***P*-value;** Control vs. Intervention<br>**(*P*<0.05)**	**Intervention group**: *Low* Risk<br>**(N=X)**	**Intervention group:** *Intermediate* Risk<br>**(N=X)**	**Intervention group:** *High* Risk<br>**(N=X)**
**ITT**	**Hospital Admission**							
	Mean length of stay	Mean (SD)	Mean (SD)	Mean (SD)	X.XX	Mean (SD)	Mean (SD)	Mean (SD)
	Total number of admissions	Median [IQR]	Median [IQR]	Median [IQR]	X.XX	Median [IQR]	Median [IQR]	Median [IQR]
	Total days of admission	Median [IQR]	Median [IQR]	Median [IQR]	X.XX	Median [IQR]	Median [IQR]	Median [IQR]
	**Outpatient Clinic Visits**							
	Total number visits	Median [IQR]	Median [IQR]	Median [IQR]	X.XX	Median [IQR]	Median [IQR]	Median [IQR]
	Additional outpatient clinic visits							
	1 additional visit	N (X %)	N (X %)	N (X %)	X.XX	N (X %)	N (X %)	N (X %)
	2 additional visits	N (X %)	N (X %)	N (X %)	X.XX	N (X %)	N (X %)	N (X %)
	3 additional visits	N (X %)	N (X %)	N (X %)	X.XX	N (X %)	N (X %)	N (X %)
**PP**	**Hospital Admission**							
	Mean length of stay	Mean (SD)	Mean (SD)	Mean (SD)	X.XX	Mean (SD)	Mean (SD)	Mean (SD)
	Total number of admissions	Median [IQR]	Median [IQR]	Median [IQR]	X.XX	Median [IQR]	Median [IQR]	Median [IQR]
	Total days of admission	Median [IQR]	Median [IQR]	Median [IQR]	X.XX	Median [IQR]	Median [IQR]	Median [IQR]
	**Outpatient Clinic Visits**							
	Total number visits	Median [IQR]	Median [IQR]	Median [IQR]	X.XX	Median [IQR]	Median [IQR]	Median [IQR]
	Additional outpatient clinic visits							
	1 additional visit	N (X %)	N (X %)	N (X %)	X.XX	N (X %)	N (X %)	N (X %)
	2 additional visits	N (X %)	N (X %)	N (X %)	X.XX	N (X %)	N (X %)	N (X %)
	3 additional visits	N (X %)	N (X %)	N (X %)	X.XX	N (X %)	N (X %)	N (X %)

Table 3. Hospital and outpatient clinic visits within 1 week

4.4 Secondary Outcomes Analysis

4.4.1 Maternal and Perinatal Adverse Outcomes

Maternal and perinatal complications will be evaluated as two predefined composite binary outcomes, as outlined in Section 2.2. The maternal composite outcome includes severe complications such as eclampsia, stroke, renal failure, pulmonary edema, HELLP syndrome, hepatic dysfunction, disseminated intravascular coagulation, placental abruption, and

maternal death. The perinatal composite outcome includes preterm birth (before 37+0 or 32+0 weeks), NICU admission, fetal growth restriction, 5-minute Apgar score, respiratory distress syndrome requiring respiratory support, and neonatal death.

Initial comparisons between the intervention and control groups will be made using chi-square tests. To further quantify group differences while accounting for potential confounding factors, multivariable logistic regression models will be applied with treatment allocation as the main independent variable and the composite outcome as the dependent variable.

These models will include adjustments for relevant clinical covariates, including gestational age at inclusion (continuous), pre-existing hypertension, antihypertensive use at baseline, body mass index (BMI), ethnicity, primigravity, and a history of severe pre-eclampsia in a previous pregnancy. Where available, additional variables may also be incorporated, such as maternal age, diabetes mellitus, autoimmune disorders (e.g. systemic lupus erythematosus, antiphospholipid syndrome), chronic kidney disease, prior placental abruption, conception via assisted reproductive technologies (e.g. oocyte donation), and smoking status.

Results will be reported as adjusted odds ratios with 95% confidence intervals and corresponding p-values. Missing data will be handled using multiple imputation, as described in Section 4.2

	Patients (N)	**Total**<br><br><br><br>**(N=X)**	**Control group**<br><br><br><br>**(N=X)**	**Intervention group**<br><br><br><br>**(N=X)**	***P*-value;** Control vs. Intervention<br><br><br>**(*P*<0.05)**	**Intervention group**: *Low* Risk<br><br><br>**(N=X)**	**Intervention group:** *Intermediate* Risk<br>**(N=X)**	**Intervention group:** *High* Risk<br><br>**(N=X)**
**ITT**	Maternal adverse Outcome	N (X %)	N (X %)	N (X %)	X.XX	N (X %)	N (X %)	N (X %)
**PP**	Maternal adverse Outcome	N (X %)	N (X %)	N (X %)	X.XX	N (X %)	N (X %)	N (X %)
**ITT**	**Maternal outcome**							
	Eclampsia	N (X %)	N (X %)	N (X %)	X.XX	N (X %)	N (X %)	N (X %)
	HELLP sydnrome	N (X %)	N (X %)	N (X %)	X.XX	N (X %)	N (X %)	N (X %)
	Stroke	N (X%)	N (X %)	N (X %)	X.XX	N (X %)	N (X %)	N (X %)
	Renal insufficiency	N (X %)	N (X %)	N (X %)	X.XX	N (X %)	N (X %)	N (X %)
	Disseminated intravascular coagulation	N (X %)	N (X %)	N (X %)	X.XX	N (X %)	N (X %)	N (X %)
	Elevated liver transaminases	N (X %)	N (X %)	N (X %)	X.XX	N (X %)	N (X %)	N (X %)
	Low platelets	N (X %)	N (X %)	N (X %)	X.XX	N (X %)	N (X %)	N (X %)
	Transfusion of blood products required	N (X %)	N (X %)	N (X %)	X.XX	N (X %)	N (X %)	N (X %)
	Pulmonary edema	N (X %)	N (X %)	N (X %)	X.XX	N (X %)	N (X %)	N (X %)
	Pulmonary embolism							
	Placental abruption	N (X %)	N (X %)	N (X %)	X.XX	N (X %)	N (X %)	N (X %)
	Maternal death	N (X %)	N (X %)	N (X %)	X.XX	N (X %)	N (X %)	N (X %)
	**Labor Outcomes**							
	Spontaneous	N (X %)	N (X %)	N (X %)	X.XX	N (X %)	N (X %)	N (X %)
	Induced	N (X %)	N (X %)	N (X %)	X.XX	N (X %)	N (X %)	N (X %)
	Pre-labor caesarean section	N (X %)	N (X %)	N (X %)	X.XX	N (X %)	N (X %)	N (X %)
	Pre-term delivery < 37 weeks	N (X %)	N (X %)	N (X %)	X.XX	N (X %)	N (X %)	N (X %)
	Pre-term delivery < 32 weeks	N (X %)	N (X %)	N (X %)	X.XX	N (X %)	N (X %)	N (X %)
	**Indication for induction of CS**							
	Maternal hypertension not controlled by maximum therapy	N (X %)	N (X %)	N (X %)	X.XX	N (X %)	N (X %)	N (X %)
	Maternal hematological abnormality	N (X %)	N (X %)	N (X %)	X.XX	N (X %)	N (X %)	N (X %)
	Maternal biochemical abnormality	N (X %)	N (X %)	N (X %)	X.XX	N (X %)	N (X %)	N (X %)
	Fetal distress	N (X %)	N (X %)	N (X %)	X.XX	N (X %)	N (X %)	N (X %)
**ITT**	Perinatal adverse outcome	N (X %)	N (X %)	N (X %)	X.XX	N (X %)	N (X %)	N (X %)
**PP**	Perinatal adverse outcome	N (X %)	N (X %)	N (X %)	X.XX	N (X %)	N (X %)	N (X %)
**ITT**	**Neonatal Outcomes**							
	Dysmaturity	N (X %)	N (X %)	N (X %)	X.XX	N (X %)	N (X %)	N (X %)
	Birth Weight	Mean (SD)	Mean (SD)	Mean (SD)	X.XX	Mean (SD)	Mean (SD)	Mean (SD)
	5-min Apgar Score	Median [IQR]	Median [IQR]	Median [IQR]	X.XX	Median [IQR]	Median [IQR]	Median [IQR]
	NICU admission	N (X %)	N (X %)	N (X %)	X.XX	N (X %)	N (X %)	N (X %)

Table 2. Maternal, Labor and Perinatal Outcomes

4.4.2. Pre-eclampsia Diagnosis

The number of participants who develop pre-eclampsia, as defined by the ISSHP 2018 criteria (2), will be compared between the intervention and control groups. Outcomes will be reported both as:

- Pre-eclampsia diagnosed within 7 days after testing
- Total incidence of pre-eclampsia at any time during follow-up (antenatal and postpartum)

Unadjusted comparisons between groups will be made using the chi-square test, or Fisher's exact test if expected cell counts are low. Results will be presented in tabular form, including absolute numbers and percentages by study arm. All results will be showed in table 3.

To further evaluate the effect of the intervention while accounting for baseline differences, a multivariable logistic regression model will be constructed, with treatment allocation as the primary independent variable. The model will adjust for relevant covariates as described in Section 4.4.1.

Patients (N)	**Total** (N=X)	**Control group** (N=X)	**Intervention group** (N=X)	***P*-value;** Control vs. Intervention (*P*<0.05)	**Intervention group**: *Low* Risk (N=X)	**Intervention group:** *Intermediate* Risk (N=X)	**Intervention group:** *High* Risk (N=X)
Pre-Eclampsia < 7 days	N (X %)	N (X %)	N (X %)	X.XX	N (X %)	N (X %)	N (X %)
Total Pre-Eclampsia	N (X %)	N (X %)	N (X %)	X.XX	N (X %)	N (X %)	N (X %)
Time to diagnosis of pre-eclampsia in those diagnosed, days	Median [IQR]	Median [IQR]	Median [IQR]	X.XX	Median [IQR]	Median [IQR]	Median [IQR]

Table 3. Pre-eclampsia Outcomes

In addition, a time-to-event analysis will be performed to assess differences in timing of pre-eclampsia diagnosis between groups. Kaplan–Meier curves will be plotted to visualize cumulative incidence over time, with comparisons made using the log-rank test. A Cox proportional hazards model will be used to estimate adjusted hazard ratios, using the same covariates as specified in the logistic regression model.

Figure 3. Kaplan Meier Curve

4.4.3. Quality of Life

Maternal quality of life will be measured using the EQ-5D-5L questionnaire, which captures self-reported health across five domains: mobility, self-care, usual activities, pain/discomfort, and anxiety/depression**.** The questionnaire will be administered at baseline and subsequently every two weeks until six weeks postpartum. EQ-5D-5L scores will be converted into utility values using the Dutch tariff, and QALYs will be calculated using the area-under-the-curve approach. (17)

Changes in EQ-5D-5L scores over time will be analyzed using repeated-measures ANOVA or linear mixed-effects models, depending on data completeness and distribution. These analyses will evaluate whether the intervention influences quality of life and productivity throughout the study period.

4.4.4. Cost-Effectiveness Analysis

The cost-effectiveness analysis will be conducted from a societal perspective, incorporating both direct medical costs and indirect costs related to productivity loss. The time horizon for the analysis will run from inclusion until six weeks postpartum.

Direct medical costs will include hospital admissions, outpatient visits, diagnostic procedures (e.g., sFlt-1/PlGF and PCr tests), and telemonitoring. Cost estimates for telemonitoring will be based on data from the SAFE@HOME study, which involved a comparable population of pregnant women using a digital remote monitoring platform. (19) All unit cost estimates will follow Dutch standard reference prices, as outlined by the Dutch Healthcare Authority (NZa) and the Dutch costing manual for health economic evaluations. (20,21)

Productivity costs will be assessed using the iPCQ questionnaire. (18) This instrument quantifies productivity loss due to health problems in both paid and unpaid work, including absenteeism and presenteeism.

Effectiveness will be assessed using the Quality-Adjusted Life Years (QALY), which incorporates both the quantity and quality of life. Quality of life will be measured using the EQ-5D-5L questionnaire, which captures self-reported health across five domains: mobility, self-care, usual activities, pain/discomfort, and anxiety/depression**.** The questionnaire will be administered at baseline and subsequently every two weeks until six weeks postpartum. EQ-5D-5L scores will be converted into utility values using the Dutch tariff, and QALYs will be calculated using the area-under-the-curve approach (17)

Multiple Imputation (MI) according to the Multivariate Imputation by Chained Equations (MICE) algorithm with 10 iterations will be used.

Incremental Cost-Effectiveness Ratios (ICERs) will be calculated to compare the cost per QALY gained between the intervention and control groups. Bootstrapping with 1,000 replications will be used to estimate 95% confidence intervals for both cost and effectiveness measures.

The results will be visualised using cost-effectiveness planes and cost-effectiveness acceptability curves.

Cost component	**Control group** (mean, € [95% CI])	**Intervention group** (mean, € [95% CI])	**Mean difference (Δ)**
Direct medical costs			
Hospital admission	XXX (CI)	XXX (CI)	XXX
Outpatient visits	XXX (CI)	XXX (CI)	XXX
sFlt-1/PlGF ratio test	XXX (CI)	XXX (CI)	XXX
Telemonitoring	0	XXX (CI)	XXX
**Total direct medical costs**	XXX (CI)	XXX (CI)	XXX
Indirect costs (productivity loss, iPCQ-based)	XXX (CI)	XXX (CI)	€XXX
**Total societal costs**	XXX (CI)	XXX (CI)	XXX
Effectiveness outcomes			
QALYs (mean per patient)	X.XX (CI)	X.XX (CI)	X.XX
**ICER (€ per QALY gained)**	-	-	XXX

Table 4. Cost-Effectiveness Analysis

4.4.5. Budget Impact Analysis

A budget impact analysis (BIA) will be conducted to assess the financial consequences of implementing the sFlt-1/PlGF ratio test combined with telemonitoring in routine obstetric care in the Netherlands. The analysis will be performed from three perspectives: societal, healthcare provider, and health insurance payer. The primary time horizon will be one year, with additional projections at three and five years to assess the longer-term financial implications.

The BIA will include the same cost components described in Section 4.4.4: direct medical costs (including biomarker testing, telemonitoring logistics, hospital stays, outpatient visits, and diagnostics) and indirect costs due to productivity losses, as captured using the iPCQ

questionnaire. Cost parameters will be sourced from national data, such as NZa tariffs, hospital-level costing information, and the Dutch costing manual for economic evaluations.

Scenario analyses will be performed to model different levels of intervention uptake (e.g., 30%, 50%, 70%) and regional variability in resource use. Sensitivity analyses will further explore how variations in key cost drivers—such as telemonitoring unit costs, test prices, and hospitalization rates—affect the overall budget impact.

4.5 Exploratory Analyses: Pre-eclampsia Incidence by Risk Category

An exploratory analysis will be conducted to assess the prognostic performance of the risk stratification model based on the sFlt-1/PlGF ratio and protein-to-creatinine (PCr) ratio. Although the model was applied only for clinical decision-making in the intervention arm, biomarker values were collected for all participants, enabling a combined analysis across both study groups.

Participants will be classified into three predefined risk categories—low, intermediate, and high—according to the protocol-defined algorithm integrating sFlt-1/PlGF and PCr values. The cumulative incidence of pre-eclampsia during follow-up will be calculated for each risk group.

Differences in incidence between the risk groups will be evaluated using the Cochran-Armitage test for trend to assess whether the likelihood of developing pre-eclampsia increases with higher risk classification. This analysis aims to explore the ability of the biomarker-based model to stratify patients by pre-eclampsia risk, independent of its role in guiding follow-up.

5. Data Management and Monitoring

Study data will be collected and managed using Castor Electronic Data Capture (EDC), a secure, web-based application designed for clinical research. Each participant will be assigned a unique study ID, which is automatically generated and unrelated to any identifiable information such as name, initials, or date of birth. All study data will be stored and analyzed using this pseudonymized identifier.

Data entry will be performed by authorized study personnel and monitored for completeness, accuracy, and internal consistency. Built-in validation rules and periodic data monitoring will support data quality assurance throughout the study period. Access to coded data will be restricted to the study team, designated monitors, quality assurance officers, and members of the Data Safety Monitoring Board (DSMB).

A separate, secure key file linking participant identifiers to study codes will be maintained exclusively by the local investigator and stored in a restricted-access location. Personal data will be handled in accordance with the General Data Protection Regulation (GDPR; Dutch: Algemene Verordening Gegevensbescherming, AVG). All study records will be retained for a minimum of 15 years, in compliance with applicable national and institutional regulations.

Monitoring activities will be conducted in accordance with Good Clinical Practice (GCP) guidelines and applicable regulatory standards to ensure the quality and integrity of the trial, as well as the safety of all participants. Site monitoring in the Netherlands will be coordinated and carried out by internal monitors from the Leiden University Medical Center (LUMC), following a pre-established monitoring plan. The frequency and scope of monitoring visits will be tailored to the needs of the study and the risk profile of participating sites, with particular

attention to informed consent procedures, data accuracy, protocol compliance, and the timely reporting of adverse events.

6. Interim Analysis

An interim evaluation will be conducted once approximately 235 participants (≈50% of the target sample size) have been enrolled. The purpose of this evaluation is to assess the feasibility and safety of the study procedures, including the rate of pre-eclampsia-related healthcare utilization during the first week after inclusion in both study arms.

This evaluation will not serve as a formal futility or efficacy analysis. No predefined statistical stopping boundaries are set. The goal is to provide the Data Safety Monitoring Board (DSMB) with blinded aggregated data to assess ongoing feasibility, participant safety, and operational implementation. The absence of a statistically significant effect will not be interpreted as grounds for early termination.

The evaluation will be conducted by an independent statistician, and results will be provided to the DSMB in blinded format. The DSMB may choose to unblind the results at its discretion.

Additionally, the DSMB will review interim safety data once 100 participants have completed full follow-up. Summaries of Serious Adverse Events (SAEs) will be provided after every 20 SAEs.

Based on its review, the DSMB may advise the sponsor to:

- Continue the trial as planned;
- Modify the sample size or study procedures;

- Or, in the case of a clinically relevant safety issue or a clear and meaningful difference in primary outcomes, recommend early termination of the study.

All DSMB decisions will be based on cumulative safety data, trial integrity, and expert clinical judgment.

7. Discussion

The PREPARE II trial builds on previous observational and interventional research evaluating the role of the angiogenic biomarker sFlt-1/PlGF in the management of suspected pre-eclampsia. As a multicentre randomized controlled trial, PREPARE II investigates whether a risk-based approach—combining protein-to-creatinine ratio (PCr), sFlt-1/PlGF testing, and telemonitoring—can enhance clinical decision-making, reduce unnecessary hospital admissions, and improve cost-effectiveness.

This Statistical Analysis Plan (SAP) was developed to ensure methodological transparency and guide the analysis of the trial's primary and secondary endpoints. The SAP defines a comprehensive analytical strategy to evaluate clinical effectiveness, safety, and economic outcomes across both study arms, and includes exploratory analyses to assess the prognostic utility of the biomarker-based risk stratification model.

Findings from the PREPARE II study may inform future clinical guidelines and healthcare policies regarding the use of angiogenic biomarkers and digital health tools in obstetric care. If successful, this strategy could reduce the burden of unnecessary monitoring and admissions for pregnant women while improving allocation of resources in maternity care syst

8. Reference List

1. Say L, Chou D, Gemmill A, Tunçalp Ö, Moller AB, Daniels J, et al. Global causes of maternal death: a WHO systematic analysis. Lancet Glob Health. 2014;2(6).

2. Brown MA, Magee LA, Kenny LC, Karumanchi SA, McCarthy FP, Saito S, et al. Hypertensive Disorders of Pregnancy: ISSHP Classification, Diagnosis, and Management Recommendations for International Practice. Hypertension. 2018 Jul 1;72(1):24–43.

3. Wang W, Xie X, Yuan T, Wang Y, Zhao F, Zhou Z, et al. Epidemiological trends of maternal hypertensive disorders of pregnancy at the global, regional, and national levels: a population-based study. BMC Pregnancy Childbirth. 2021 Dec 1;21(1).

4. Magee LA, Brown MA, Hall DR, Gupte S, Hennessy A, Karumanchi SA, et al. The 2021 International Society for the Study of Hypertension in Pregnancy classification, diagnosis & management recommendations for international practice. Pregnancy Hypertens. 2022 Mar 1;27:148–69.

5. Chappell LC, Cluver CA, Kingdom J, Tong S. Pre-eclampsia. Lancet. 2021 Jul 24;398(10297):341–54.

6. Hagmann H, Thadhani R, Benzing T, Karumanchi SA, Stepan H. The promise of angiogenic markers for the early diagnosis and prediction of preeclampsia. Clin Chem. 2012 May;58(5):837–45.

7. Zhang J, Klebanoff MA, Roberts JM. Prediction of adverse outcomes by common definitions of hypertension in pregnancy. Obstetrics and gynecology. 2001;97(2):261–7.

8. Delahaije DHJ, Smits LJM, Van Kuijk SMJ, Peeters LL, Duvekot JJ, Ganzevoort W, et al. Care-as-usual provided to formerly preeclamptic women in the Netherlands in the next pregnancy: health care consumption, costs and maternal and child outcome. Eur J Obstet Gynecol Reprod Biol. 2014;179:240–5.

9. Stevens W, Shih T, Incerti D, Ton TGN, Lee HC, Peneva D, et al. Short-term costs of preeclampsia to the United States health care system. Am J Obstet Gynecol. 2017 Sep 1;217(3):237-248.e16.

10. Vatish M, Strunz-McKendry T, Hund M, Allegranza D, Wolf C, Smare C. sFlt-1/PlGF ratio test for pre-eclampsia: an economic assessment for the UK. Ultrasound Obstet Gynecol. 2016 Dec 1;48(6):765–71.

11. H Z, M H, S V. The sFlt-1:PlGF Ratio in Women with Suspected Preeclampsia. New England Journal of Medicine. 2016 May 5;374(18):1785–6.

12. Cerdeira AS, O'Sullivan J, Ohuma EO, Harrington D, Szafranski P, Black R, et al. Randomized Interventional Study on Prediction of Preeclampsia/Eclampsia in Women With Suspected Preeclampsia: INSPIRE. Hypertension. 2019 Oct 1;74(4):983–90.

13. Duhig KE, Myers J, Seed PT, Sparkes J, Lowe J, Hunter RM, et al. Placental growth factor testing to assess women with suspected pre-eclampsia: a multicentre, pragmatic, stepped-wedge cluster-randomised controlled trial. Lancet. 2019 May 4;393(10183):1807–18.

14. Wind M, van den Akker-van Marle ME, Ballieux BEPB, Cobbaert CM, Rabelink TJ, van Lith JMM, et al. Clinical value and cost analysis of the sFlt-1/PlGF ratio in addition to the

spot urine protein/creatinine ratio in women with suspected pre-eclampsia: PREPARE cohort study. BMC Pregnancy Childbirth. 2022 Dec 1;22(1).

15. Schulz KF, Altman DG, Moher D. CONSORT 2010 statement: Updated guidelines for reporting parallel group randomized trials. Ann Intern Med. 2010 Jun 1;152(11):726–32.

16. Bartsch E, Medcalf KE, Park AL, Ray JG. Clinical risk factors for pre-eclampsia determined in early pregnancy: systematic review and meta-analysis of large cohort studies.

17. Versteegh M, M. Vermeulen K, M. A. A. Evers S, de Wit GA, Prenger R, A. Stolk E. Dutch Tariff for the Five-Level Version of EQ-5D. Value Health. 2016 Jun 1;19(4):343–52.

18. Bouwmans C, Krol M, Severens H, Koopmanschap M, Brouwer W, Roijen LH Van. The iMTA Productivity Cost Questionnaire: A Standardized Instrument for Measuring and Valuing Health-Related Productivity Losses. Value in Health. 2015 Sep 1;18(6):753–8.

19. van den Heuvel JFM, van Lieshout C, Franx A, Frederix G, Bekker MN. SAFE@HOME: Cost analysis of a new care pathway including a digital health platform for women at increased risk of preeclampsia. Pregnancy Hypertens. 2021 Jun 1;24:118–23.

20. Kanters TA, Bouwmans CAM, Van Der Linden N, Tan SS, Hakkaart-van Roijen L. Update of the Dutch manual for costing studies in health care. PLoS One. 2017 Nov 1;12(11):e0187477.

21. Nederlandse Zorgautoriteit [Internet]. [cited 2025 Mar 24]. Available from: https://puc.overheid.nl/nza/

Appendix 1. EQ-5D-5L Questionnaire

Appendix 2. i-PCQ Questionnaire
