# Supplementary material for: Cost-effectiveness of the sFlt-1/PlGF ratio and telemonitoring in managing suspected pre-eclampsia: protocol for the PREPARE II randomised controlled trial
Source: BMJ Open. 2026 Jul 2;16(7):e113516. doi: 10.1136/bmjopen-2025-113516 (PMC13331053; doi:10.1136/bmjopen-2025-113516)
Supplement: online supplemental file 3 [file bmjopen-16-7-s003.pdf]

Information letter PREPARE II study

Research about better care and safe reduction of hospital stays for women with (possible) pre-eclampsia

Introduction – PREPARE II Study

Dear Madam,

You receive this information letter because your doctor thinks you may develop pre-eclampsia (pregnancy poisoning). Pre-eclampsia is diagnosed when you have high blood pressure, protein in your urine, a small baby, or complaints such as headache, flashing lights in your vision, or pain/pressure in your upper belly. These complaints can appear before the illness is certain. At this moment, we do not know if you will really develop pre-eclampsia.
In this study we want to see if, by using an extra blood test and home blood pressure monitoring, we can better predict which women have a higher risk of problems from pre-eclampsia, and which women can safely continue care at home.

The aim of this study is to improve care and reduce hospital admissions. You decide yourself if you want to join. Participation is voluntary.

In this letter you will read:

- what participation means,
- what is expected from you,
- the possible advantages and disadvantages,
- and how we protect your privacy.
-

If you decide to join, we will ask you to fill in and sign the consent form (Appendix E). Please take your time to read this information carefully. Do you have questions? You can ask your doctor or the independent expert. Their contact details are at the end of this letter.

What does this mean for you?
In this letter you can read what the study is about, what it means for you, and what the possible benefits and risks are. We understand this is a lot of information. Please take your time to read it. If you decide to join, you can sign the consent form (Appendix E).

Ask your questions
When you decide, you can use this letter, but we also advise you to:

- Ask questions to the researcher who gave you this information.
- Discuss the study with your partner, family, or friends.
- Contact the HELLP Foundation, the patient group for women who had (pre-)eclampsia.
- Ask the independent expert, [redacted]
- Read the information on www.rijksoverheid.nl/mensenonderzoek.

1. General information

The Leiden University Medical Center (LUMC) started this study. We call LUMC the “sponsor.” Researchers (doctors, nurses, research staff) carry out the study. We need 470 participants for this study.The Medical Ethics Committee Leiden–The Hague–Delft has approved this study.

2. Background

Pre-eclampsia affects about 2–5% of all pregnancies. It is a condition that can become serious and can cause problems for both mother and baby. The main signs of pre-eclampsia are high blood pressure and protein in the urine. In some cases, it can lead to damage to organs of the mother, such as the liver or kidneys. Sometimes the baby grows less well because the placenta does not work properly. In some cases, the baby needs to be born earlier. This can cause low birth weight or premature birth.

In the Netherlands, women with suspected pre-eclampsia are often admitted to the hospital or have frequent check-ups at the outpatient clinic. During these visits, the doctor monitors you carefully, for example with blood pressure checks and blood or urine tests. Later it often turns out that these hospital stays or extra visits were not necessary, because the women did not become seriously ill. Still, these extra appointments or hospital stays can be difficult for the patient and are also expensive for healthcare.

With the current tests, doctors cannot always predict which women will really become sick. This makes it hard to decide who needs extra care and who can safely go home. Earlier research (the PREPARE I study) showed that a new blood test, the **sFlt-1/PlGF ratio**, can help. When this test shows a normal value, the chance of developing pre-eclampsia within one week is very small. This test can help doctors estimate which women need extra care and which women can safely go home.

3. What is the aim of this study?

In this study, we want to see if a combination of two tests – the protein/creatinine ratio in your urine and the sFlt-1/PlGF ratio in your blood – together with the right care (for example hospital admission, home monitoring, or standard check-ups) can reduce unnecessary hospital stays and outpatient visits.

We want to check if this way of risk assessment and guidance is safe, and at the same time can help to reduce care where possible.

Earlier research showed that women with a normal result on this blood test almost never develop pre-eclampsia within one week. Therefore, we expect that using this test does not give extra risk for participants, also not the risk of missing pre-eclampsia or problems caused by it.

4. How does the study work?

Step 1: Are you suitable to join?
Your doctor thinks you may have pre-eclampsia. This suspicion comes when you have high blood pressure together with one or more other complaints or signs.

Possible complaints or signs of pre-eclampsia are:

- Protein in the urine
- Slower growth of the baby
- Headache
- Seeing stars or flashing lights
- Nausea or vomiting
- Pain between the shoulder blades
- Pain or a tight feeling in the upper belly
- Sudden swelling of face, hands, or feet

You can join this study if you:

- are between 20 and 37 weeks pregnant,
- are pregnant with one baby (not twins), and
- have high blood pressure with at least one of the complaints above.

Step 2: What care do you get during the study?
If you join the study, you are placed by lottery in one of two groups. You will be in group 1 (control group) or group 2 (study group). Both you and your doctor know your group.

Group 1 – Control group

The result of the blood test (sFlt-1/PlGF ratio) is **not** used for your care. You get the usual medical care, the same as if you did not join the study.

- Your doctor makes decisions based on:
  - your blood pressure,
  - the amount of protein in your urine (the PCR),
  - your complaints,
  - the progress of your pregnancy,
  - and the growth of your baby.

This is done according to the normal hospital guidelines. You can find more information about this standard care in Appendix D.

Group 2 – Study group (intervention group)
In this group, the results of the extra blood test (sFlt-1/PlGF ratio) and urine test (protein/creatinine ratio) are used to estimate your risk of pre-eclampsia. Based on the results, you are placed in one of three risk groups.

Each group has its own type of care:

Low risk (PCR < 30 and sFlt-1/PlGF ratio < 38):
You do not need extra check-ups or hospital stay. You stay under the care of your midwife or doctor as planned. If you get new or worse complaints, you must always contact your care provider again.

Medium risk (PCR ≥ 30 or sFlt-1/PlGF ratio ≥ 38):
You get one extra check at the hospital and you will use LUMC@HOME for pregnant women (telemonitoring).

This telemonitoring package includes a blood pressure monitor that you use at home to measure your blood pressure every day. You will also use an app (the LUMC@HOME care app) to report if you have any symptoms.

To use this telemonitoring, you need to be registered as a patient at LUMC. For this, personal and medical data needed for your registration and care will be shared with LUMC, such as your name, date of birth, citizen service number (BSN), and insurance details.

We only use this information to register you and to guide your care as a patient. We do not use this information for research.

The information you enter is reviewed on working days by the PREPARE II team at the Leiden University Medical Center. If there are any concerns, they will contact your doctor.

During the weekend, your data is not checked automatically. You will receive clear instructions explaining when you should contact the hospital yourself. These instructions can be found in Appendix E.

Before you start using LUMC@HOME, you will receive instructions on how to use the devices. The blood pressure monitor used for telemonitoring is available at the hospital where you receive care.

High risk (PCR ≥ 30 and sFlt-1/PlGF ratio ≥ 38):
You are directly admitted to the hospital for extra monitoring and care.

This division into groups follows a fixed schedule (see Appendix C). Your doctor will explain which group you are in and which care belongs to it.

Step 3: What happens during the study?

When your doctor thinks you may develop pre-eclampsia, you will get a blood test. An extra tube of blood (about 20 ml) is taken. In this blood, the sFlt-1/PlGF ratio is measured.
All participants get this blood test, but only in the study group the result is used for your care.
We also ask you to fill in a digital questionnaire every two weeks until six weeks after birth. The questions are about:

- your daily life,
- your work,
- and how you feel (your mood).

If you find digital tools or Dutch language difficult, you can discuss this with the researcher. The information is also available in English or sometimes in another language you prefer. If needed, the questionnaires can also be given on paper.

What is different from normal care?
In this study, some parts of your care may be different.

If you are in group 1 (control group):
Your care changes very little. One extra tube of blood is taken, and we ask you to fill in a short questionnaire every two weeks until six weeks after birth. The last questionnaire, six weeks after birth, is important to finish the study.
The rest of your care is the same as usual, according to hospital guidelines (see Appendix D).

If you are in group 2 (study group):
Your care is based on the results of the extra blood and urine tests. This is different from normal care, where these tests are not standard.

Depending on the results:

- you may get telemonitoring with telemonitoring package and one extra hospital visit,
- you may be admitted to the hospital for extra observation,
- or you may follow your normal care plan without extra visits.
  In this group, you also fill in a questionnaire every two weeks until six weeks after birth.

So, your care may be different from normal, but we always follow a safe plan for your situation. Your doctor will discuss this with you.

5. What agreements do we make with you?

We want the study to go well. That is why we make the following agreements with you:

- You come to every appointment.
- You or someone close to you contacts the researcher in these situations:
  - You are admitted or treated in another hospital than LUMC.
  - You suddenly get health problems, including new or worse complaints.
  - You do not want to continue in the study.
  - Your phone number, address, or email address changes.

6. What side effects or problems can you experience?

There are very few risks or side effects with this study.

- For the blood test, one extra tube of blood (about 20 ml) is taken. This can give a short unpleasant feeling, like a prick or a small bruise.
- If you are in the telemonitoring group, we ask you to measure your blood pressure every day and fill in complaints in an app. Some women may find this time-consuming or tiring.
- We also ask you to fill in a digital questionnaire every two weeks until six weeks after birth. This takes about ten minutes each time.

You will not receive medicines and you will not get treatments that are not medically needed. Your own doctor stays responsible for your care and can always decide to change the study plan if that is better for your health or for your baby.

7. What are the benefits and risks of joining the study?

Joining the study has both advantages and disadvantages. You can take your time to think and also discuss this with your partner, family, or care provider.

Benefits

- You may need fewer hospital visits or no hospital stay if your risk of pre-eclampsia is low.
- Your care is based on extra information from blood and urine tests, so unnecessary check-ups or admissions may be avoided.
- You help scientific research that may improve care for pregnant women with risk of pre-eclampsia in the future.

Disadvantages and possible risks

- The combined test (blood and urine) gives a good estimate of the risk of pre-eclampsia. But like all tests, it is not 100% reliable. It is possible that you still develop pre-eclampsia, even with a low or medium result, and need to be admitted.
- Earlier research (PREPARE I study) showed that women with normal results on both tests almost never got pre-eclampsia within one week. Less than 2 in 100 women developed pre-eclampsia, and none of them had serious complications for mother or baby.
- It remains very important that you always contact your doctor or the research team if you have complaints, or if existing complaints get worse.
- If you are in the medium-risk group, we ask you to:
    - measure your blood pressure every day at home,
    - fill in your complaints in the app,
    - and fill in digital questionnaires (about 10 minutes each time) until six weeks after birth.

This takes time and effort. If you find digital tools or Dutch difficult, you can discuss this with your doctor. Other options are possible, like paper forms or help with filling in.

Finally: your doctor may always change the study plan if needed for medical reasons, for example if your complaints suddenly get worse or test results show more risk. Your health and your baby's health always come first.

Do you not want to join?
You decide yourself if you join. Participation is voluntary. If you do not want to join, you get the standard care for your pregnancy.

If you join, you can always change your mind and stop, also during the study. You do not have to say why. You must tell the researcher if you stop. The data already collected will still be used for the study. If you want, any stored body material can be destroyed.

8. When does the study stop?

The researcher will tell you if there is new information about the study that is important for you. Then you can decide if you want to continue.

Your participation in the study stops:

- At the standard follow-up visit 6 weeks after birth.
- If you decide to stop yourself.
- If the sponsor, the government, or the ethics committee decides to stop the study.

The whole study ends when all participants are finished. After all data are analysed, the researcher will send you an email with the main results. This will be about 2–3 years after your participation.

9. What happens after the study?

After the study, all collected data are analysed. Your normal medical care continues as usual with your midwife or doctor.

Your personal data will be kept confidential and anonymised before being used in scientific publications. In these publications, it will not be possible to identify you.

If you want, you can receive a summary of the main results after the study. You can indicate this in Appendix G of this letter.

If you have questions or comments after the study, you can always contact the research team. Their contact details are at the end of this letter.

10. What do we do with your data and body material?

If you join the study, you also give permission to collect, use, and store your data.

Which data do we keep?
We keep:

- your name
- your sex
- your ethnicity
- your address
- your date of birth
- your health information
- medical data collected during the study. These may also include data from earlier tests during this pregnancy, such as the non-invasive prenatal test (NIPT).

These data are stored for 15 years, as required by law for medical research.

What happens with my blood?
During the study, blood is taken to do the test that is needed. This blood is not automatically stored for future research. For storing an extra tube of blood in the Biobank for future research, you will get a separate information letter and consent form (PIF). You decide if you want to take part. This is separate from the current study and fully voluntary.

Why do we collect, use, and store your data?
We use your data to answer the research questions and to publish results in scientific journals.

Use of NIPT data
In addition to the main study, some participants may take part in extra scientific research using data from the non-invasive prenatal test (NIPT) that you had earlier in your pregnancy. This concerns general characteristics of the DNA in your blood. We will use these data for research purposes only.

Use of placental tissue after birth
After the birth, any remaining placental tissue may be used for additional scientific research. This will only take place after the baby is born. The use of placental tissue will not affect the birth or the care for you and your baby.
You do not have to do anything for this additional research. No extra blood samples, tests, or hospital visits are needed. The use of these data and the placental tissue will not affect your treatment or your baby's treatment.

Permission for child data
We may also collect medical data about your baby, for example birth weight, any complications, and possible admission to the neonatal intensive care unit (NICU). Because this is child data, the law requires permission from the other parent or legal guardian. That is why we ask your partner to sign a separate consent form (Appendix F).

How do we protect your privacy?
Your data get a code. Only this code is used on documents, not your name. The key to the code is kept safe in the hospital. This way, your data can only be linked to you by authorised persons. In reports and publications, it is not possible to identify you.

Who can see your data?
Some people may see your name and other personal data without the code. These are:

- Monitors, who check if the study is done well and safely, for example for the sponsor (LUMC).
- National and international authorities, such as the Dutch Health and Youth Care Inspectorate (IGJ), who by law may access your data without your permission.
- Members of the committee that checks the safety of the study (for example, a Data Safety Monitoring Board).
- The researcher of the sponsor, who needs your contact details to send the questionnaires.

All these people must keep your data secret. Your permission is asked for access, except where the law makes an exception (for example the IGJ).

What happens with unexpected findings?
During the study we may find something not related to the study, but important for your health or your baby's health. This is called an unexpected finding. If this happens, we discuss it with you and, if needed, with your doctor. Together you decide if further testing or treatment is needed. You choose what to do.

Can you withdraw your permission for data use?
Yes, you can withdraw your permission at any time. This applies for this study and for other research.
But: if researchers already collected data, they may still use them. For your body material, researchers will destroy it after you withdraw permission. If tests were already done, the results may still be used.

Do you want more information about your privacy?

- For your rights about data protection, see: www.autoriteitpersoonsgegevens.nl
- Questions or complaints? Contact the organisation responsible for your data: LUMC (see Appendix A).

- If you have a complaint, you can first talk to the research team. You can also read the LUMC privacy statement (see Appendix A), or contact the LUMC Data Protection Officer.
- You can also make a complaint at the Dutch Data Protection Authority.

Where can you find more information about the study?
On www.zorgevaluatienederland.nl you can find more information. No personal data will be shared there.

11. Do you get a reimbursement if you join the study

The extra tests for the study cost you nothing. You will not be paid for joining this study. Any extra travel or parking costs you make because of the study will be reimbursed. You can ask the research team for more information about this.

12. Are you insured during the study?

For everyone who joins this study, insurance is arranged through LUMC. This insurance covers harm that you may get as a result of the study, but only under certain conditions.

Please note: not all harm is covered. Harm from risks that are already explained in this information letter is not covered by the insurance.

In Appendix B you can read more about what is and is not covered, and who you can contact if you have damage.

13. We inform your general practitioner and/or midwife

If you take part in this study, we will send your GP and/or midwife a short letter. This letter only says that you are joining the PREPARE II study. This is important so your care providers know about your situation and can follow your pregnancy well. Your GP or midwife will not receive research results. Only if it is medically needed, specific information will be shared.

14. Do you have questions?

Questions about the study? You can ask the research team.

Do you want advice from someone independent, who has no interest in the study? You can contact the independent expert (see Appendix A). She knows a lot about the study, but she is not part of the study team.

Do you have a complaint? You can talk with the researcher or your treating doctor. If you do not want this, you can go to the complaints committee of the hospital (see Appendix A).

15. How do you give permission for the study?

First, take your time to think about this study. After that, you tell the researcher if you understand the information and if you want to join or not.

Do you want to join? Then you fill in the consent form that comes with this letter. Both you and the researcher will get a signed copy of this consent form.

Thank you for your time.

Kind regards,

[redacted]

Attachments to this information

A. Contact details
B. Information about the insurance
C. Overview of the study protocol (diagram)
D. Information about standard care for pre-eclampsia
E. Information about *The Box* for pregnant women
F. Consent form(s)
G. Form to receive information about study results

Appendix A: Contact details

If you have questions about the content of the study, you can call:

Principal investigator:

[redacted]

Independent Expert:

[redacted]

Coordinating Researcher:

[redacted]

Coordinating Researcher:

[redacted]

Coordinating Researcher:

[redacted]

[redacted]

Appendix B: Information about the insurance

LUMC has arranged an insurance for everyone who takes part in the study. The insurance pays for harm that you get because you joined the study. It covers harm that happens during the study, or within 4 years after the study. You must report the harm within 4 years to the insurer.

The insurance pays a maximum of:

- €650,000 per person
- €5,000,000 for the whole study
- and €7,500,000 per year for all studies from the same sponsor.

Please note: the insurance does not cover the following harm:

- Harm from a risk that was explained to you in this letter.
  (Except if the risk was bigger than expected, or if the risk was very unlikely.)
- Harm to your health that would have happened even if you did not join the study.
- Harm that happens because you did not follow instructions (or not correctly).
- Harm to the health of your children or grandchildren.
- Harm from a treatment method that already exists, or from research about an existing treatment.

Appendix C: Schematic overview of study protocol

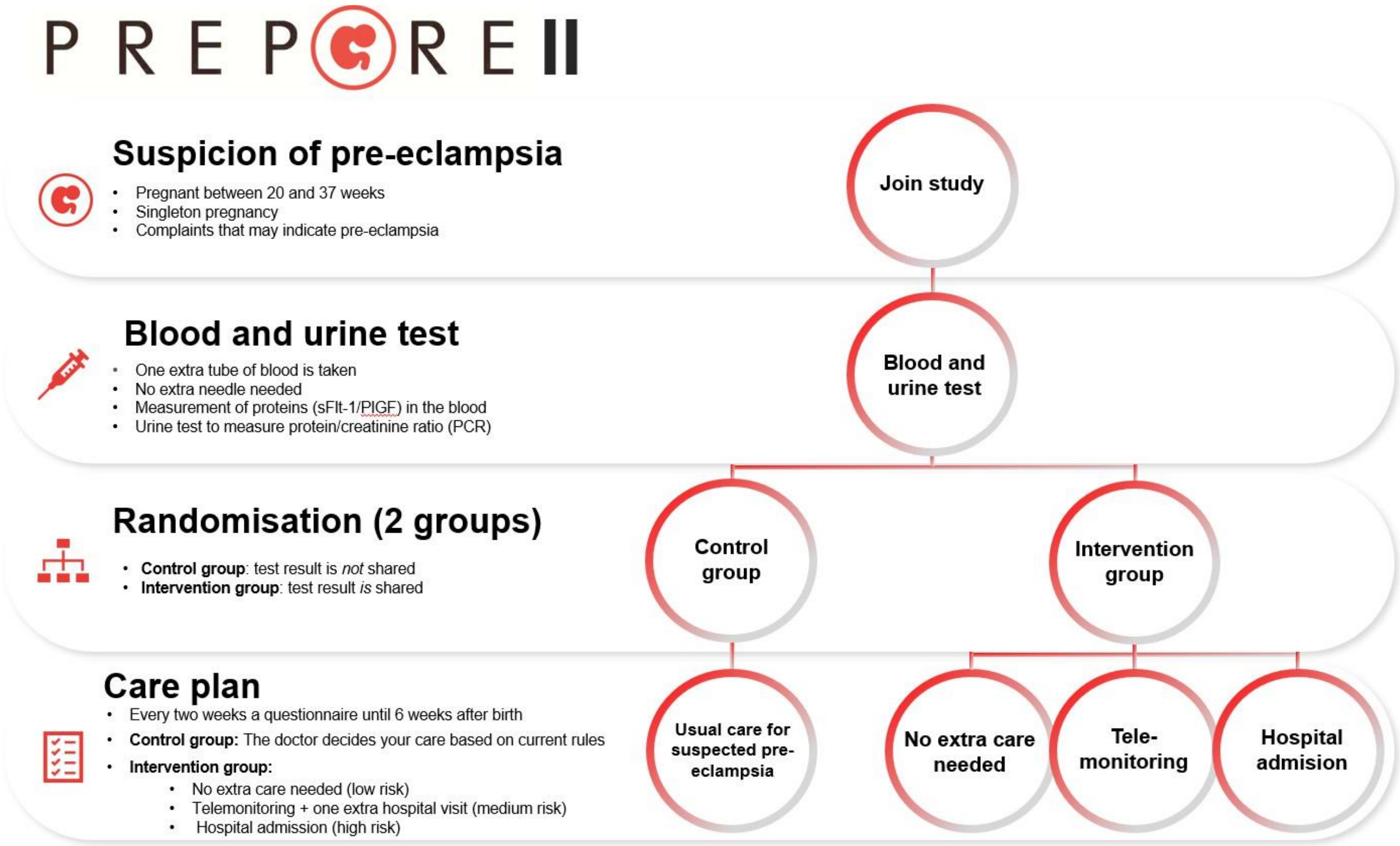

Appendix D – Standard care in case of suspected pre-eclampsia

If you do not take part in this study, or if you are in group 1 (control group), the care for pre-eclampsia will follow the usual hospital procedures.

The doctor will check your situation based on several factors:

- your blood pressure
- the amount of protein in your urine (protein/creatinine ratio – PCR)
- your complaints and the course of the pregnancy
- the growth of your baby

Based on this information, the doctor decides if extra monitoring, hospital admission, or additional treatment is needed.

This decision is made by a hospital doctor (specialist care), following the international ISSHP guideline and the Dutch national guideline on hypertensive disorders in pregnancy. More information can be found at the Dutch guideline database: Richtlijnendatabase – Hypertensieve aandoeningen in de zwangerschap. [1,2]

Appendix E: Consent Form Participant PREPARE II

- I have read the information letter. I was able to ask questions. My questions were answered well. I had enough time to decide if I want to join.
- I know that participation is voluntary. I also know that I can decide at any moment to stop taking part in the study. I do not need to give a reason if I want to stop.
- I give the researcher permission to inform my general practitioner/midwife that I take part in this study.
- I give permission that information about me, my pregnancy and delivery, and about my child can be collected from my midwifery practice or from other hospitals, if I or my child are admitted to another hospital during this study.
- I give the researchers permission to collect and use my data and/or body material. The researchers do this only to answer the research question of this study.
- I give permission to share my contact details with the researcher from the coordinating center for sending the questionnaires.
- I know that some people may look at my data to check the study. These people are listed in the information letter. I give them permission to see my data for this check.
- I give permission to store my study data and my child's data for 15 years after the end of this study.

Please tick yes or no in the table below

I give permission to store my body material for future research about pre-eclampsia.	Yes ☐	No ☐
I give permission to register my ethnicity in the study data.	Yes ☐	No ☐
I give permission to be contacted after this study to ask if I want to take part in a follow-up study.	Yes ☐	No ☐
I give permission to request my NIPT data.	Yes ☐	No ☐

☐ I want to take part in this study.

My name (participant) ………………………………..

Signature: ……………………… Date : __(dd) / __(mm) / __(yy)

I declare that I have fully informed this participant about the mentioned study. If new information becomes available during the study that may affect the participant's consent, I will inform the participant in time.

Name of researcher (or representative):……………………………….

Signature: ……………………… Date : __(dd) / __(mm) / __(yy)

The participant will receive a complete information letter together with a signed copy of this consent fo

Appendix F: Consent Form Partner

Why this form?

In this study, we also collect data about your child (for example, pregnancy outcomes, delivery information, and neonatal care). According to the law, this requires the consent of both parents with parental authority. Your partner (the pregnant woman) has already agreed to participate. With this form, we also request your consent.

What data will be collected?

- Pregnancy and delivery outcomes
- Information about your child's health at and after birth
- Any additional examinations and/or hospital admissions of your child

This information will be treated confidentially and used only for scientific research within PREPARE II.

Voluntariness

- Your consent is entirely voluntary.
- You may withdraw your consent at any time without giving a reason.
- Withdrawing consent will have no consequences for the care provided to your partner or your child.

Declaration by partner/parent with parental authority

I have read and understood the information about the PREPARE II study.
I agree to the collection and use of my child's data as described above.
Name of partner/parent: **..**

Signature: .... Date: ....

For the researcher

I confirm that I have sufficiently informed the partner/parent about this consent form and have given the opportunity to ask questions.
Name of researcher: **..**

Signature: .... Date: ....

Appendix G – Information about study results

After the PREPARE II study, you can receive a summary of the main results. This information will be available once the data has been analyzed and the results have been published.

Would you like to be informed about the results after the study ends? Please tick what applies to you:
☐ Yes, I would like to receive a summary of the main results after the study.
☐ No, I do not want to receive information about the study results.

If you choose "Yes," you will receive the summary by e-mail or by post, as soon as it is available.

Please indicate your preference below:

☐ By e-mail: E-mail address:

You can sign this appendix together with the consent form and give it to your healthcare provider.

References

1. Magee LA, Brown MA, Hall DR, Gupte S, Hennessy A, Karumanchi SA, et al. The 2021 International Society for the Study of Hypertension in Pregnancy classification, diagnosis & management recommendations for international practice. Pregnancy Hypertens. 2022;27:148-69.
2. NVOG. *Hypertensieve aandoeningen in de zwangerschap* [Internet]. Utrecht: Nederlandse Vereniging voor Obstetrie en Gynaecologie (NVOG); 2019 [cited 2026 May 7]. Available from: Richtlijnendatabase – Hypertensieve aandoeningen in de zwangerschap
