# Supplementary material for: Cost-effectiveness of the sFlt-1/PlGF ratio and telemonitoring in managing suspected pre-eclampsia: protocol for the PREPARE II randomised controlled trial
Source: BMJ Open. 2026 Jul 2;16(7):e113516. doi: 10.1136/bmjopen-2025-113516 (PMC13331053; doi:10.1136/bmjopen-2025-113516)
Supplement: Supplementary file 4 [file bmjopen-2025-113516.reviewer_comments.pdf]

PEER REVIEW HISTORY

BMJ Open publishes all reviews undertaken for accepted manuscripts. Reviewers are asked to complete a checklist review form and are provided with free text boxes to elaborate on their assessment. These free text comments are reproduced below.

ARTICLE DETAILS

Title (Provisional)

Cost-effectiveness of the sFlt-1/PlGF ratio and telemonitoring in managing suspected pre-eclampsia: protocol for the PREPARE II randomized controlled trial

Authors

Wind, Merlijn; Peters, Aniek; Akker-van Marle, Elske M; Vanden Auweele, Kim; van Bodegom - Vos, Leti; Boekhorst, Ferry; Cobbaert, Christa; Hermes, W.; Van Hoorn, Marion E; Van Den Haak, Lukas; Lutke Holzik, Margo; Van Wyk, Linda; Tsonaka, Roula; Teng, Y.K Onno; Sueters, M.

VERSION 1 - REVIEW

**Reviewer**	**1**
**Name**	**Di Simone , Nicoletta**
**Affiliation**	**Humanitas University**
**Date**	**07-Feb-2026**
**COI**	**None**

This paper describes the methodology and setting-up of an impressive project (RCT across 6 centers) regarding the role of the sFlt-1/PlGF ratio in reducing healthcare costs in the management of women with suspected pre-eclampsia between 20 and 37 weeks.

Please expand on the long term cardiovascular complications in women who experienced preeclampsia during pregnancy in the Introduction (you can refer to : Inversetti A, Pivato CA, Cristodoro M, Latini AC, Condorelli G, Di Simone N, Stefanini G. Update on long-term cardiovascular risk after pre-eclampsia: a systematic review and meta-analysis. Eur Heart J Qual Care Clin Outcomes. 2023 Nov 16:qcad065. doi: 10.1093/ehjqcco/qcad065. Epub ahead of print. PMID: 37974053.

Overall, the manuscript is clearly written and the RCT is well designed.

**Reviewer**	**2**
**Name**	**Castro-Sánchez, Adelaida María**
**Affiliation**	**Universidad de Almeria, Enfermería, Fisioterapia y Medicina**
**Date**	**23-Apr-2026**

COI **None**

The manuscript entitled “Cost-effectiveness of the sFlt-1/PlGF ratio and telemonitoring in managing suspected pre-eclampsia: protocol for the PREPARE II randomized controlled trial” presents a robust and novel study. The protocol is well conceived and appropriately designed. In addition, the large sample size is a major strength that is likely to support the external validity of the findings. The manuscript is suitable for publication, subject to the following minor comments:

The authors should clarify whether results will be expressed as incremental cost-effectiveness ratios (ICERs), reflecting the additional cost per additional unit of outcome achieved.

Further detail is needed on the cost-effectiveness acceptability curve, specifically how it will represent the probability that each alternative is cost-effective across different willingness-to-pay thresholds per Quality-Adjusted Life Year.

The authors should specify the assumed or reference value of a QALY in the Netherlands.

A sensitivity analysis should be included to assess the robustness of the findings to variations in key parameters, assumptions, and methodological approaches.

VERSION 1 - AUTHOR RESPONSE

Reviewer #1

Dr. Nicoletta Di Simone , Humanitas University

Comment

This paper describes the methodology and setting-up of an impressive project (RCT across 6 centers) regarding the role of the sFlt-1/PlGF ratio in reducing healthcare costs in the management of women with suspected pre-eclampsia between 20 and 37 weeks.
Please expand on the long term cardiovascular complications in women who experienced preeclampsia during pregnancy in the Introduction.

Response

We thank the reviewer for the positive and encouraging feedback on our manuscript and for this valuable suggestion. We agree that the long-term cardiovascular consequences of pre-eclampsia are important and should be acknowledged in the Introduction We have therefore added a brief statement on the increased long-term cardiovascular risk associated with pre-eclampsia. (Inversetti et al., 2023).

Changes made

- Lines 79-81: “Pre-eclampsia remains a significant global health concern, affecting 3–5% of pregnancies and posing both immediate and long-term cardiovascular risks to mothers, as well as significant risks to the unborn child.”

Reviewer #2

Dr. Adelaida María Castro-Sánchez, Universidad de Almeria

Comment

The manuscript entitled *"Cost-effectiveness of the sFlt-1/PlGF ratio and telemonitoring in managing suspected pre-eclampsia: protocol for the PREPARE II randomized controlled trial"* presents a robust and novel study. The protocol is well conceived and appropriately designed. In addition, the large sample size is a major strength that is likely to support the external validity of the findings. The manuscript is suitable for publication, subject to the following minor comments:

- The authors should clarify whether results will be expressed as incremental cost-effectiveness ratios (ICERs), reflecting the additional cost per additional unit of outcome achieved.
- Further detail is needed on the cost-effectiveness acceptability curve, specifically how it will represent the probability that each alternative is cost-effective across different willingness-to-pay thresholds per Quality-Adjusted Life Year. The authors should specify the assumed or reference value of a QALY in the Netherlands.
- A sensitivity analysis should be included to assess the robustness of the findings to variations in key parameters, assumptions, and methodological approaches.

Response

We thank the reviewer for the positive and encouraging feedback on our manuscript and for these valuable suggestions. We agree that further clarification of the economic evaluation strengthens the methodological transparency of the study.

- We chose not to present incremental cost-effectiveness ratios (ICERs), as ICERs can be unstable and difficult to interpret when differences in costs or effects are small. In addition, ICERs do not adequately reflect uncertainty in decision-making. Instead, we use a net-benefit approach and present cost-effectiveness acceptability curves (CEACs), which are widely recommended as a more informative and robust method for presenting uncertainty in economic evaluations.

- We have further expanded the description of the CEAC and specified the willingness-to-pay thresholds commonly applied in the Netherlands (€20,000–€80,000 per QALY).

- In addition, we have included sensitivity analyses to assess the robustness of the findings to variations in key assumptions and methodological choices.

Changes made:

- Lines 352-358: "In a net benefit analysis, costs will be related to QALYs and summarised in a cost-effectiveness acceptability curve. This curve plots, for each willingness-to-pay threshold per QALY gained, the probability that an intervention
yields a higher net monetary benefit than the comparator. The range of thresholds will

include values commonly applied in the Netherlands (€20,000–€80,000 per QALY), allowing decision makers to assess cost-effectiveness within the nationally accepted willingness-to-pay framework."

- Added the following references:
    - Smulders YM, Thijs A. *[The cost per year of life gained: trends and internal contradictions].* Ned Tijdschr Geneeskd. 2006;150(45):2467– 70.
    - Raad voor de Volksgezondheid en Zorg (RVZ)/National Council for Public Health and Health Care. *Sustainable and Meaningful Care [in Dutch: Zinnige en Duurzame Zorg].* The Hague, The Netherlands; 2006.

- Lines 358-359: "Sensitivity analyses will be conducted to assess the robustness of the findings to variations in key parameters, assumptions, and methodological choices."
